# Decoding IGLL5 Mutation‐Mediated BCR Signaling: A Novel Mechanism of CD8^+^ T Cell Exhaustion and Ocular MALT Lymphoma Progression

**DOI:** 10.1002/advs.202518780

**Published:** 2026-05-12

**Authors:** Andi Zhao, Haoyu Wei, Chenyu Zhou, Xinping Sun, Shuyan Da, Haiyu Liu, Zijin Wang, Hui Zhu, Shiya Shen, Qing Shao, Qi Gong, Hu Liu, Xuejuan Chen

**Affiliations:** ^1^ Department of Ophthalmology The First Affiliated Hospital with Nanjing Medical University Nanjing China; ^2^ Nanjing Medical University Nanjing China

**Keywords:** B‐cell receptor (BCR) signaling, IGLL5 mutations, OAML (ocular adnexal MALT lymphoma), T‐cell exhaustion

## Abstract

Ocular adnexal mucosa‐associated lymphoid tissue lymphoma (OAML) is driven by both genetic and immune microenvironmental factors, yet its pathogenic mechanisms remain incompletely understood. We previously identified *IGLL5* as a recurrently mutated gene associated with poor prognosis in OAML. Functional and mechanistic analyses focusing on the S47G and A54G mutants show that these variants enhance association with the CD79A/CD79B complex, leading to persistent B‐cell receptor (BCR) signaling. This signaling is accompanied by upregulation of CXCL10 and CXCL11, increased CD8^+^ T cell recruitment, and an exhaustion‐associated dysfunctional phenotype that may contribute to an immune‐tolerant microenvironment. Pharmacologic inhibition further shows that combined BTK inhibitor and rituximab treatment suppresses IGLL5‐associated BCR activation. Together, these findings support a mutation‐associated mechanism in a subset of OAML and nominate IGLL5‐related signaling as a potential therapeutic vulnerability.

## Graphical abstract

OAML harbors recurrent IGLL5 mutations that reinforce CD79A/CD79B‐associated BCR signaling. Mechanistic analysis of the S47G and A54G variants reveals induction of CXCL10/CXCL11, enhanced CD8^+^ T‐cell recruitment, and exhaustion‐associated dysfunction, supporting an immune‐tolerant niche. Combined BTK inhibition and rituximab suppress this mutant‐associated signaling axis, highlighting a therapeutically actionable vulnerability in a subset of OAML.

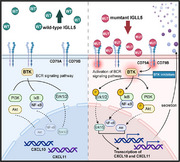

## 1 Introduction

Orbital and ocular adnexal lymphomas are the most common primary orbital malignancy in adults, with ocular adnexal marginal zone lymphoma of mucosa‐associated lymphoid tissue (MALT) subtype (OAML) as the most frequent subtype [1, 2]. OAML primarily affects the ocular regions, including the conjunctiva, eyelids, and lacrimal gland, and poses a significant clinical risk due to its potential for malignant progression and recurrence [3]. The etiology of MALT is complex, involving chronic inflammatory stimuli that may arise from microbial infections or autoimmune disorders, which are believed to drive lymphomagenesis through persistent immune activation [4]. Unique to OAML, specific genetic and chromosomal abnormalities, such as the t(11;18)(*BIRC3*/*MALT1*) and t(14;18)(*IgH*/*MALT1*) translocations, though common in other MALT lymphomas, are infrequent in OAML. Recent investigations utilizing next‐generation sequencing (NGS) have highlighted a distinct genetic landscape for OAML, characterized by mutations in genes like *TNFAIP3*, *MYD88*, and various histone modifiers (*CREBBP*, *KMT2D*, *TBL1XR1*). These mutations are known to activate the NF‐κB pathway and disrupt immune surveillance [5]. The distinct mutational landscape of OAML may reflect unique biological characteristics that set it apart from other MALT types. Notably, the *IGLL5* gene has emerged as a newly identified mutation in OAML, adding another layer to its genetic profile and underlining the genetic heterogeneity that distinguishes OAML from other MALT lymphomas [6].

*IGLL5* emerged as a frequently mutated gene in several high‐grade B‐cell neoplasms despite the limited reports of *IGLL5* mutations in various MALT lymphomas other than OAML. Notably, studies have identified *IGLL5* mutations in aggressive lymphomas such as vitreoretinal lymphoma (VRL) [7, 8, 9], diffused large B‐cell lymphoma (DLBCL) [10, 11], Burkitt lymphoma [12], multiple myeloma [13], chronic lymphocytic leukemia (CLL) [14], and follicular lymphoma [15, 16]. Furthermore, *IGLL5* mutations have been associated with poorer progression‐free survival (PFS) and overall survival (OS), suggesting their potential impact on treatment outcomes [10, 17]. These findings suggest a potential specificity of *IGLL5* alterations in OAML compared to other MALT sites. The functional implications of *IGLL5* mutations may involve alterations in B‐cell signaling pathways, contributing to tumorigenesis. However, it remains unclear why these genetic changes are associated with B‐cell lymphoma and the precise mechanisms by which they facilitate the development of lymphoma at a molecular level.

A recent study has indicated that *IGLL5* may influence lymphoma pathology by affecting *MYC* expression [18]. Despite this, no studies have specifically aimed to identify genetic lesions associated with *IGLL5* mutations in lymphoma or to describe the transcriptomic profiles of malignant cells, particularly in OAML. Thus, the precise molecular mechanisms underlying *IGLL5*’s oncogenic signaling and its role in the activation of carcinogenic pathways remain unresolved. In this study, we aim to provide the first comprehensive examination of the transcriptomic characteristics of OAML harboring *IGLL5* mutations. To elucidate the oncogenic actions of various *IGLL5* mutations, we also investigate the downstream targets associated with *IGLL5* mutant oncogenic signaling. Furthermore, we identify evidence of CD8^+^ T cell exhaustion within the tumor microenvironment of OAML, suggesting a complex interplay between *IGLL5* mutations and immune evasion in this malignancy.

## 2 Results

### 2.1 WES Analysis Identifies IGLL5 as a Diagnostic Marker in B‐Cell Lymphoma

**FIGURE 1:**
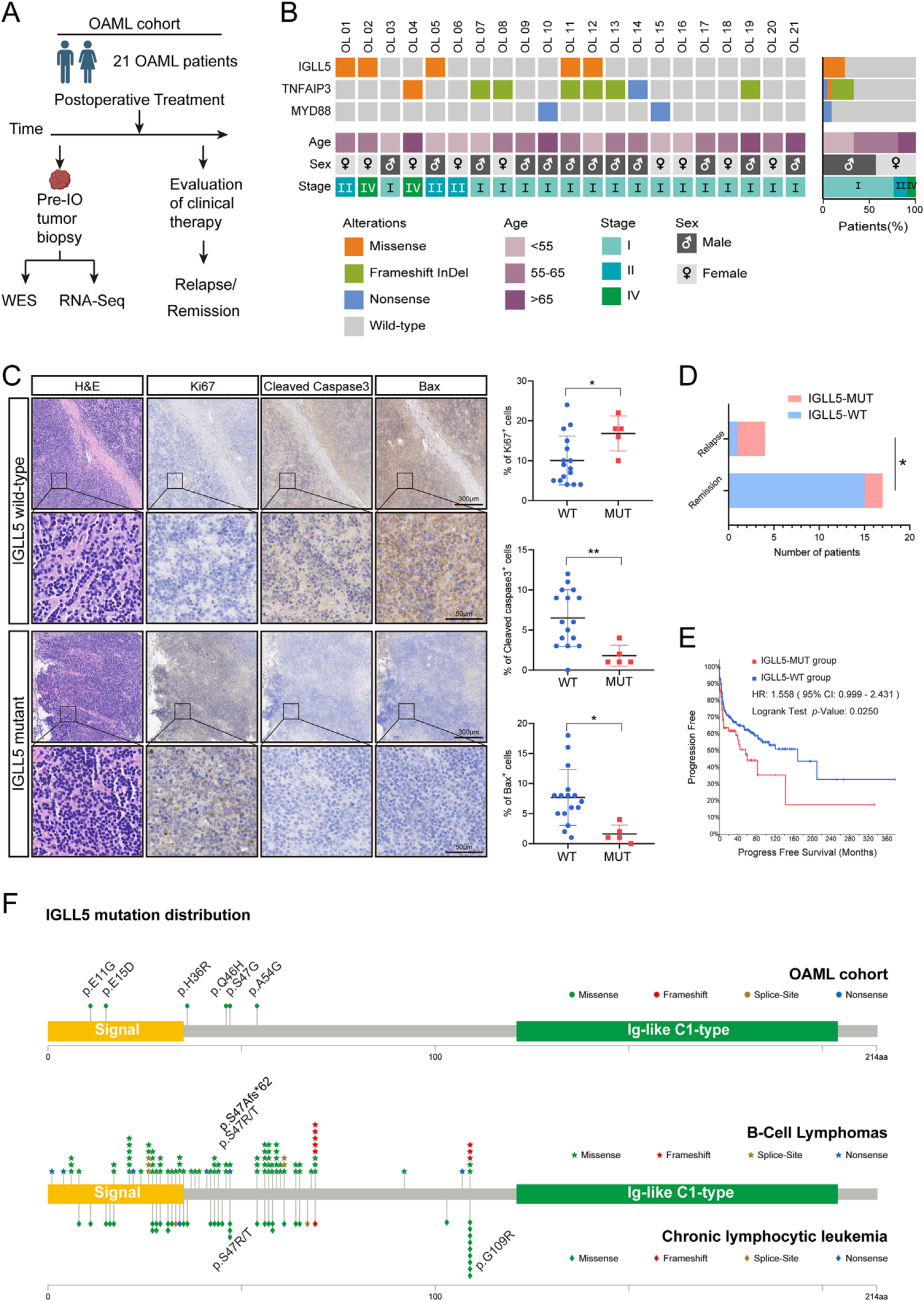
IGLL5 Mutations Are Associated With the Development and Prognosis of B‐cell Lymphomas. (A) Workflow of the ocular adnexal marginal zone lymphoma (OAML) cohort. Tumor samples from patients were resected and analyzed using Whole‐Exome Sequencing (WES) and transcriptomic profiling. Patients then underwent postoperative treatment, with disease status monitored throughout follow‐up. (B) Heatmap displaying three high‐frequency mutant genes (*IGLL5*, *TNFAIP3*, and *MYD88*) identified through WES in this OAML cohort. Each column represents an individual patient sample, while each row represents a gene or clinicopathological feature. (C) Representative histological and immunohistochemical features of lymphoma tissues with and without *IGLL5* mutation. Representative images (left) show H&E staining and immunohistochemical staining for Ki67, cleaved Caspase 3, and Bax. Quantification (right) shows the percentage of positive cells. Statistical Quantification (right) shows the percentage of positive cells. (D) Association between somatic *IGLL5* mutations and response to a rituximab (RTX)‐containing postoperative treatment in the OAML cohort. Statistical significance was determined using Fisher's exact test. (E) Kaplan‐Meier plot showing the progression‐free survival (PFS) in *IGLL5*‐mutant and wild‐type patients based on the cBioPortal for Cancer Genomics database(https://www.cbioportal.org/). Survival differences were assessed using the log‐rank test. (F) Schematic illustration of *IGLL5* mutations seen in our OAML cohort (upper) and other B‐lymphocyte malignancies from cBioPortal for Cancer Genomics, including B‐cell lymphoma (middle) and Chronic Lymphocytic Leukemia (lower). The majority of *IGLL5* mutations are missense mutations clustered mainly in N‐terminal functional regions. Statistical significance is denoted as ^*^ *p*<0.05; ^**^ *p*<0.01.

To investigate pathogenic drivers of OAML, we performed whole‐exome sequencing (WES) on tumor tissues and matched peripheral blood samples from 21 OAML patients [6], along with transcriptomic profiling via RNA sequencing (RNA‐seq) of the tumors (Figure 1A). Analysis of these data revealed recurrent mutations in known drivers such as *TNFAIP3* and *MYD88*, and notably, a previously uncharacterized high‐frequency mutation in *IGLL5* (Figure 1B). This mutation was detected in 5 out of 21 OAML cases, including one case with two mutations.

Tumors harboring *IGLL5* mutation showed upregulation of proliferation‐related genes and downregulation of pro‐apoptotic genes, suggesting a potential mechanism for their roles in OAML pathogenesis (Figure 1C). Clinically, *IGLL5* mutations were associated with poorer outcomes: three out of four relapsed patients carried *IGLL5* mutations, whereas 15 out of 17 patients who achieved remission were *IGLL5* wild‐type (Figure 1D).

Consistent with a role in B‐cell malignancy, *IGLL5* expression was enriched in mature B lymphocytes and further elevated in B‐cell lymphomas (GSE115795, Figure S1A–C) [19]. Analysis of the TCGA cohort confirmed higher IGLL5 transcription in DLBCL than in normal tissues (Figure S1D). Prognostic analysis using the cBioPortal database (https://www.cbioportal.org/) revealed that IGLL5 mutation correlated significantly with reduced progression‐free survival in B‐cell lymphoma patients, unlike mutations in *TNFAIP3* or *MYD88*, which showed no clear prognostic association in this cohort (Figure 1E; Figure S1E–H).

Across diverse B‐lymphocyte malignancies, including this OAML cohort, DLBCL, and CLL, most *IGLL5* mutations were missense variants affecting functional domains in the N‐terminal region. These alterations are predicted to disrupt normal protein function and contribute to lymphomagenesis (Figure 1F). Together, these findings strongly implicate IGLL5 mutation as a diagnostic and prognostic marker in B‐cell lymphomas.

### 2.2 IGLL5 Mutants Promote Proliferation and Reduce Apoptotic Responses in B‐Cell Lymphoma

**FIGURE 2:**
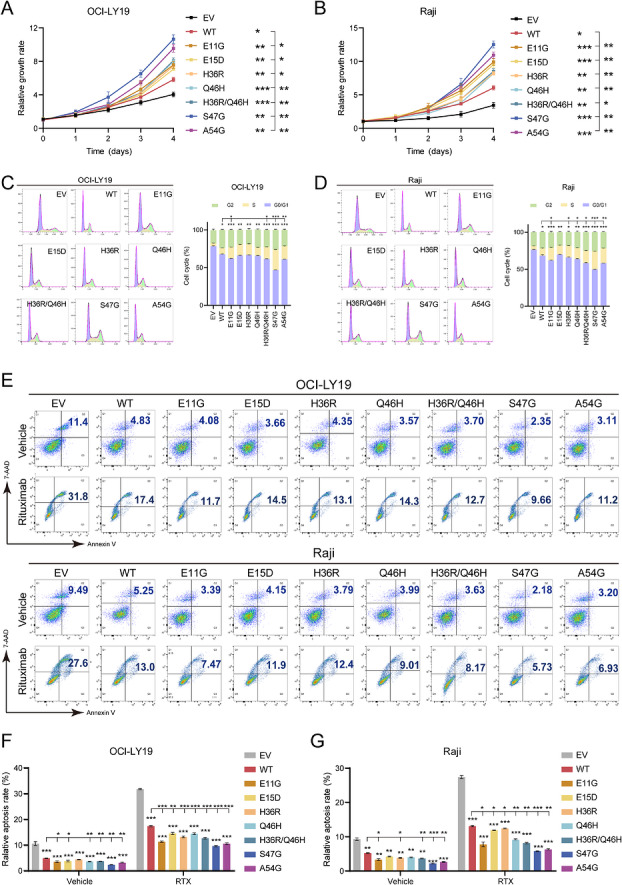
IGLL5 Mutations Promote Proliferation and Attenuate Apoptotic Responses in B‐cell Lymphoma Cells. (A, B) Proliferation potential of *IGLL5*‐mutant cells assessed by CCK‐8 assay. OCI‐LY19 (A) and Raji(B) cells transduced with empty vector (EV), wild‐type (WT) IGLL5, or specific IGLL5 mutants were cultured for 4 days, and cell viability was measured. Absorbance values were quantified to assess proliferation rates. Data are presented as mean ± SD from three biologically independent experiments, each performed in triplicate. Statistical significance was determined by one‐way ANOVA followed by Tukey's multiple‐comparisons test. Significance on the left of the vertical line is normalized to the EV control, and on the right, normalized to the WT control. (C, D) Cell cycle analysis of IGLL5 mutants performed by flow cytometry. OCI‐LY19(C) and Raji(D) cells transduced with EV, WT, or specific IGLL5 mutants. The percentage of cells in different cell cycle phases (G1, S, G2/M) was quantified. Statistical significance was determined by one‐way ANOVA followed by Tukey's multiple‐comparisons test. Significance under the horizontal line is normalized to the EV control, and above is normalized to the WT control. (E–G) Effects of IGLL5 mutations on apoptotic responses under basal conditions and after RTX treatment in vitro. OCI‐LY19(E) and Raji(F) cells stably expressing WT IGLL5 or its mutants were treated with 20 µg/mL RTX or left untreated for 1 h, and apoptosis was measured by flow cytometry. Statistical significance in (G) was determined by one‐way ANOVA followed by Tukey's multiple‐comparisons test, with significance indicated below the horizontal line normalized to the EV control and above the line normalized to the WT control. All data are presented as the means ± SD of three biologically independent experiments. Statistical significance is denoted as ^*^ *p* <0.05, ^**^ *p* <0.01, ^***^ *p* <0.001.

To assess the effects of IGLL5 mutations on the proliferative and survival properties of B‐cell lymphoma cells, OCI‐LY19 and Raji cells were examined by CCK‐8 assay and cell‐cycle analysis. All tested IGLL5 mutations promoted proliferation in both cell lines (Figure 2A, B). Cells expressing the H36R+Q46H, S47G, or A54G mutants also showed a partial reduction in the G0/G1‐phase fraction with corresponding increases in S‐phase entry (Figure 2C, D). We next evaluated apoptotic responses under basal conditions and after RTX exposure in vitro. Under untreated conditions, IGLL5‐mutant cells showed mildly reduced apoptosis relative to wild‐type controls. RTX increased apoptosis in all groups, but mutant IGLL5‐expressing cells remained less apoptotic than wild‐type cells (Figure 2E–G). Together, these data indicate that IGLL5 mutations promote proliferation and attenuate apoptotic responses in B‐cell lymphoma cells, including a reduced direct apoptotic response to RTX in vitro.

### 2.3 IGLL5 Mutants Drive OAML Progression via BCR Pathway Activation

**FIGURE 3:**
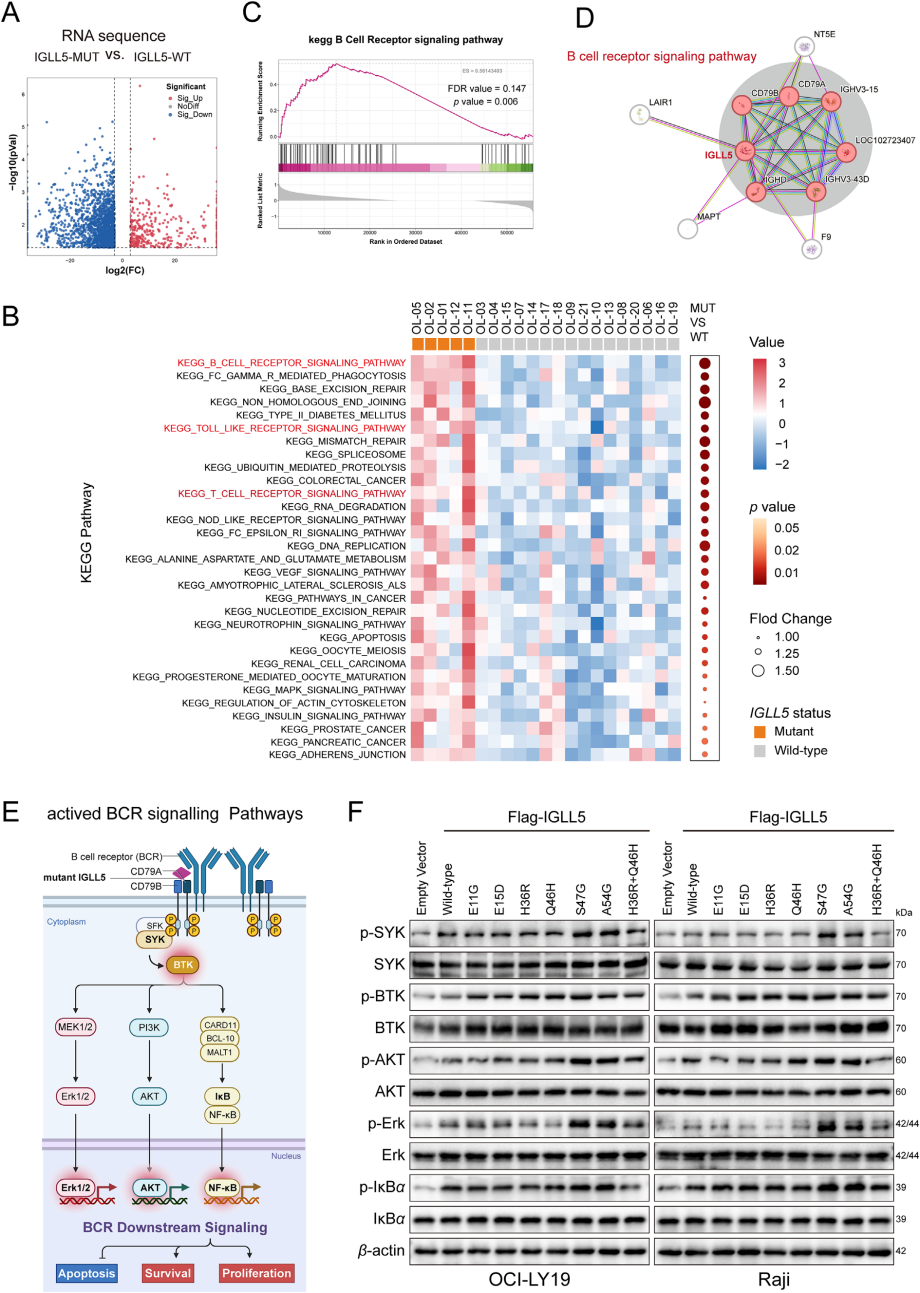
Mutations in IGLL5 Promote the Activation of the B‐cell Receptor (BCR) Pathway to Different Extents. (A) Transcriptomic profiling comparing *IGLL5*‐mutant and wild‐type OAML samples. (B) Heatmap (left) shows the differential KEGG pathways between two groups based on Gene Set Variation Analysis (GSVA). Each column represents a sample, and each row represents a pathway. The bubble plot (right) displays the fold changes and statistical significance for the indicated pathways. (C) Gene Set Enrichment Analysis (GSEA) showing enrichment of the BCR signaling pathway in the IGLL5‐mutant group. (D) STRING database(https://string‐db.org/) analysis reveals that IGLL5 interacts with key molecules, with molecules in the gray circle representing those involved in the BCR signaling pathway. (E) Diagrams illustrating the BCR signaling pathway and its key downstream pathways. (F) OCI‐LY19 and Raji cells were transduced with lentiviruses containing EV, wild‐type IGLL5, or the indicated IGLL5 mutants. Phosphorylated and total proteins were assessed by western blot to evaluate activation of BCR‐related downstream signaling pathways. Representative immunoblots from three biologically independent experiments are shown. Quantification is presented in Figure S3. Statistical significance was determined by one‐way ANOVA followed by Tukey's multiple‐comparisons test.

Based on these findings, we stratified the OAML cohort by IGLL5 mutation status and performed RNA extraction followed by transcriptome sequencing (Figure 3A). Differentially expressed genes were analyzed using Gene Set Enrichment Analysis (GSEA) and Gene Set Variation Analysis (GSVA). GSVA revealed significant enrichment of 31 pathways in the IGLL5‐mutated group compared to the wild‐type group. Notably, pathways involved in immune and inflammatory responses, including the B‐cell receptor (BCR), Toll‐like receptor (TLR), and T‐cell receptor signaling pathways, were significantly enriched (Figure 3B). Consistently, GSEA confirmed upregulation of the BCR signaling pathway in RNA‐seq data from patients with IGLL5 mutations (Figure 3C). Furthermore, the STRING database (https://string‐db.org/) predicted interactions between IGLL5 and key molecules within the BCR signaling pathway, such as CD79A and CD79B, the BCR‐associated transmembrane signaling proteins (Figure 3D). In summary, supported by prior studies [20, 21], our findings demonstrate that persistent BCR activation drives B‐cell lymphoma pathogenesis and disease progression.

We then selected two representative B‐lymphoma cell lines, OCI‐LY19 and Raji, based on previous literature [22, 23]. To model IGLL5 mutations, we first knocked out endogenous *IGLL5* in these cells. Subsequently, we transduced them with lentiviruses expressing either an empty vector, wild‐type *IGLL5*, or specific *IGLL5* mutant (E11G, E15D, H36R, Q46H, S47G, A54G, and H36R+Q46H; Figure S2A–C). To assess BCR pathway activation, we performed Western blot analysis of key downstream transcription factors and signaling molecules (BTK, SYK, ERK, AKT, and IκBα), examining both their total protein levels and phosphorylation status, as depicted in the schematic (Figure 3E). The results demonstrated that the IGLL5 mutations activated the BCR pathway to varying degrees, with the S47G and A54G mutations inducing particularly robust activation (Figure 3F and Figure S2D).

### 2.4 IGLL5 Mutants are Associated with BCR Pathway Activation Through Enhanced Interaction with CD79A/CD79B

**FIGURE 4:**
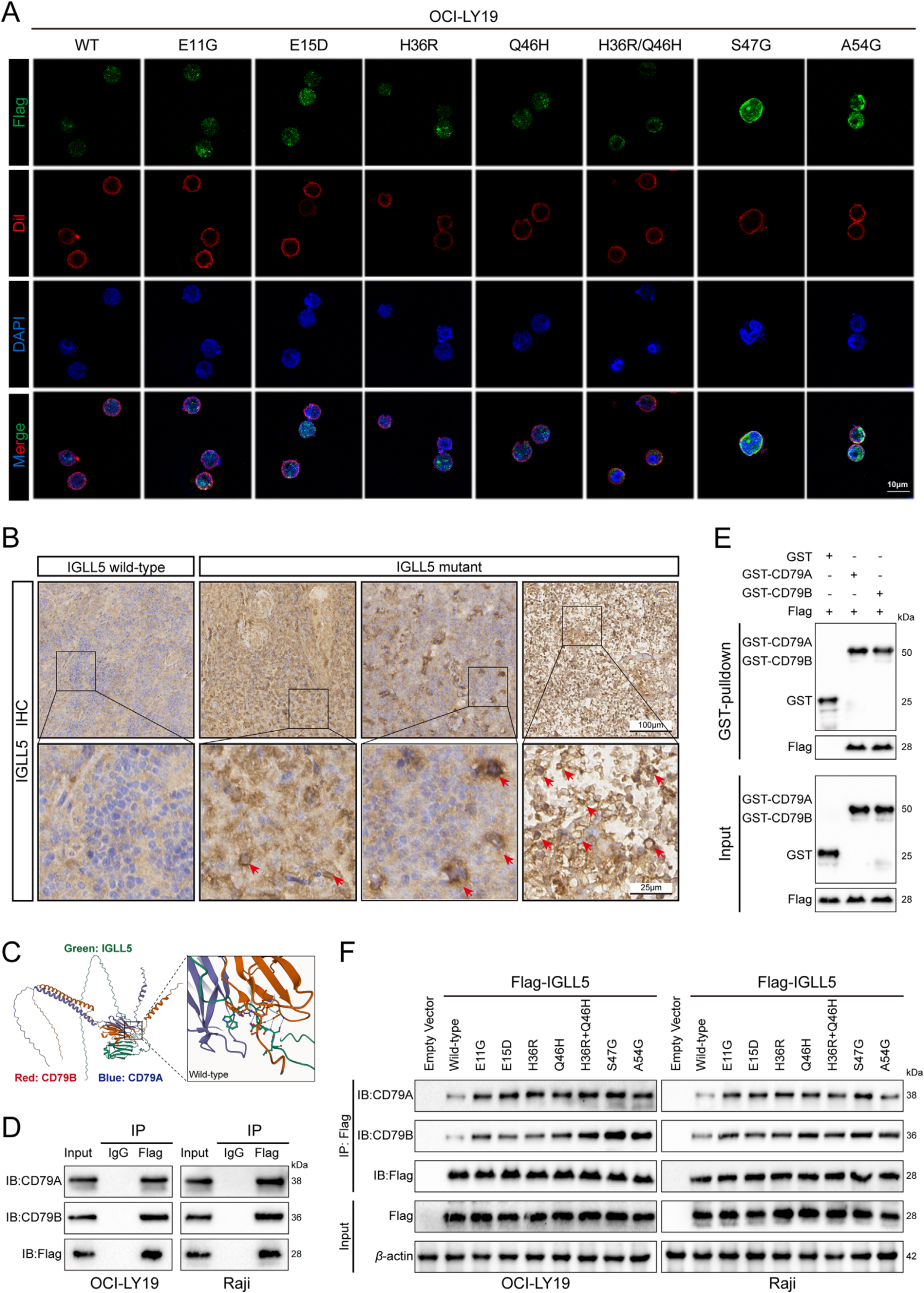
IGLL5 Mutations Result in Enhanced Binding to the CD79A/CD79B Complex. (A) Immunofluorescence analysis of the subcellular localization of wild‐type IGLL5 and indicated mutants in OCI‐LY19 cells transfected with FLAG‐tagged plasmids. Cells were fixed and stained with an anti‐FLAG antibody, followed by a goat‐anti‐rabbit IgG‐AF488 antibody (green). Nuclei were counterstained with DAPI (blue), and cell membranes were stained with Dil perchlorate (red). (B) Representative histological and immunohistochemical images showing the subcellular localization of IGLL5 variants and their distribution relative to the nucleus and membrane in OAML tissues. (C) The 3D structures of CD79A, CD79B, and wild‐type IGLL5 predicted by AlphaFold3(https://alphafoldserver.com/) demonstrate that wild‐type IGLL5 can interact with CD79A and CD79B. (D) Co‐immunoprecipitation (Co‐IP) and western blot assays in OCI‐LY19 and Raji cells showing endogenous interactions between IGLL5 and CD79A, as well as IGLL5 and CD79B. Representative immunoblots from three biologically independent experiments are shown. (E) Binding analysis of IGLL5 with CD79A and CD79B in vitro using glutathione S‐transferase (GST) pull‐down assays. Fusion protein beads were employed for the pull‐down assay, and interactions were detected by Western blot with anti‐GST and anti‐FLAG antibodies. Representative immunoblots from three biologically independent experiments are shown. (F) Co‐IP and western blot assays in OCI‐LY19 and Raji cells expressing WT or mutant IGLL5, showing interactions with CD79A and CD79B. Mutant IGLL5 showed enhanced association with CD79A and CD79B compared with WT IGLL5. Representative immunoblots from three biologically independent experiments are shown. Quantification is presented as mean ± SD from three biologically independent experiments. Statistical significance was determined by one‐way ANOVA followed by Tukey's multiple‐comparisons test.

Given our findings that IGLL5 mutations activate the BCR pathway and promote aggressive cell phenotypes, we next investigated the impact of these mutations on IGLL5 itself. First, we examined whether the mutations significantly affected *IGLL5* transcription level, which indicated no significant effect (Figure S3A,B). Since previous studies suggested IGLL5 is a secreted protein, we measured its levels in conditioned media using enzyme‐linked immunosorbent assay (ELISA). The results demonstrated that these missense mutations had no apparent effect on IGLL5 protein expression or stability (Figure S3C). We then assessed the subcellular localization of wild‐type and mutant IGLL5 using immunofluorescence. The wild‐type IGLL5 exhibited relatively equal distribution between intracellular and extracellular compartments. In contrast, mutant forms predominantly accumulated intracellularly, with pronounced aggregation on the cell membrane. Notably, the S47G and A54G mutations induced the most substantial membrane localization (Figure 4A and Figure S3D). Immunohistochemical analysis of OAML tissues further corroborated these localization patterns (Figure 4B).

Therefore, we hypothesized that mutated IGLL5 might activate the BCR pathway through interactions with membrane molecules. Building on our earlier protein interaction findings (Figure 2D), we specifically postulated that IGLL5 could associate with the BCR receptor complex, particularly CD79A/CD79B, to initiate BCR signaling. Supporting this notion, gene correlation analysis in DLBCL revealed significant positive correlations between *IGLL5* expression and both *CD79A* and *CD79B* expression (Figure S3E). To further investigate potential physical interactions between IGLL5 and CD79A/CD79B, we employed the Alphafold‐3 online protein structure prediction server to model complexes of wild‐type and mutant IGLL5 bound to CD79A and CD79B (Figure 4C and Figure S4A). Subsequently, Co‐IP experiments confirmed a direct interaction between IGLL5 and both CD79A and CD79B (Figure 4D).

Furthermore, GST pull‐down assays with western blot analysis confirmed the direct protein‐protein interaction between IGLL5 and the CD79A/CD79B complex (Figure 4E and Figure S4B). Co‐IP experiments in cells carrying the IGLL5 mutation plasmids demonstrated that all mutated forms of IGLL5 could have a stronger affinity for interaction partners CD79A/CD79B, with the S47G and A54G mutations conferred the strongest binding affinity (Figure 4F and Figure S4C). To investigate the structural basis for these observations, we employed the DynaMut webserver (http://biosig.unimelb.edu.au/dynamut/) to predict the impact of the mutations on IGLL5 stability and flexibility. The analysis indicated a destabilizing effect for several variants, including H36R, Q46H, S47G, and A54G. These mutations were predicted to increase the flexibility of the N‐terminus region, suggesting potential alterations in IGLL5's structural integrity and interaction interfaces (Figure S4D). To further assess whether mutant IGLL5‐associated BCR activation was functionally dependent on CD79A/CD79B, Raji cells expressing wild‐type, S47G, or A54G IGLL5 were transfected with siRNAs targeting CD79A and/or CD79B. Silencing of CD79A and CD79B significantly reduced phosphorylation of key BCR downstream effectors in cells expressing mutant IGLL5, whereas total protein levels remained largely unchanged (Figure S4E), supporting that mutant IGLL5‐associated BCR activation is functionally dependent on CD79A/CD79B. Collectively, these results indicate that IGLL5 mutations alter subcellular localization, increase plasma membrane accumulation, enhance association with CD79A/CD79B, and promote BCR‐related signaling.

### 2.5 BTK Inhibitor Suppresses IGLL5 Mutation‐Associated Malignant Phenotypes in Lymphoma Cells

**FIGURE 5:**
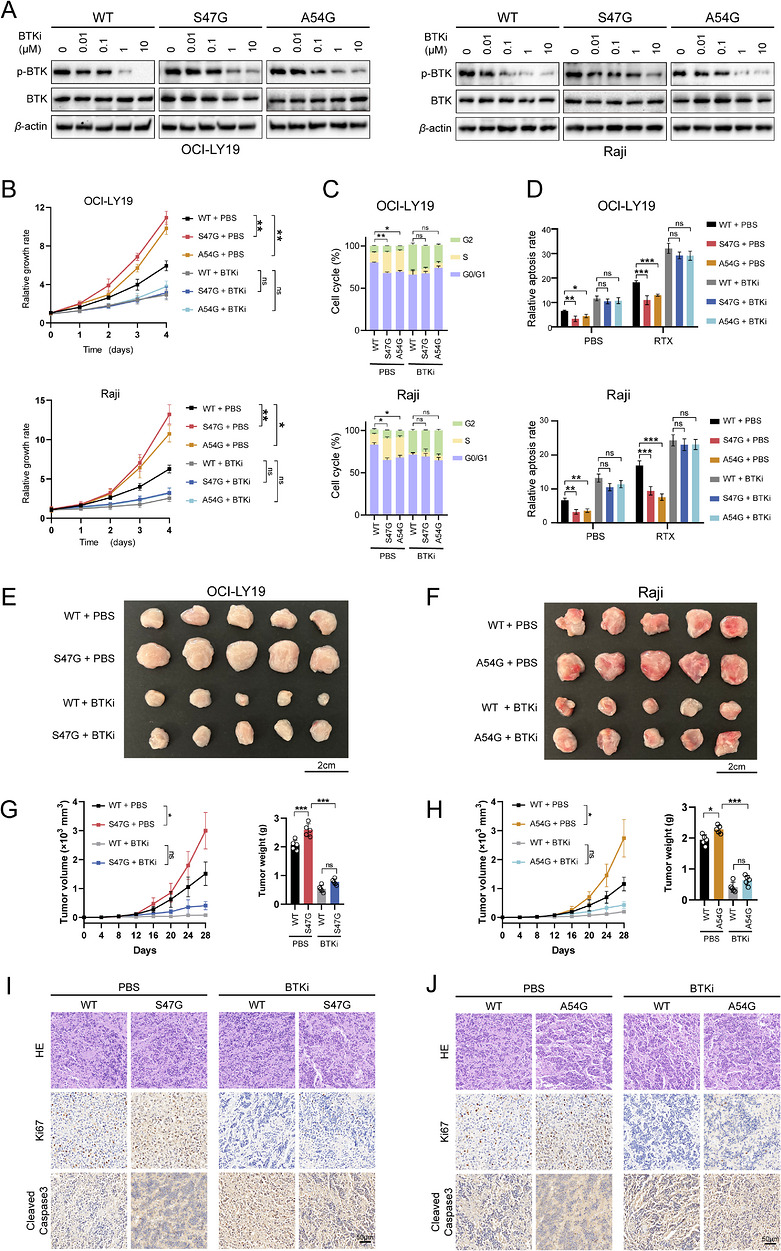
BTKi Inhibits Growth and Induces Apoptosis in IGLL5‐Mutant Lymphoma Cells. (A) Wild‐type, S47G‐mutant, and A54G‐mutant OCI‐LY19 and Raji cells were treated with increasing concentrations of the BTK inhibitor (BTKi) ibrutinib for 2 h. Phospho‐BTK and total BTK protein levels were assessed by Western blotting. Representative immunoblots from three biologically independent experiments are shown. Quantification is presented in Figure S5A. (B) Proliferation of wild‐type, S47G‐, and A54G‐mutant OCI‐LY19 and Raji cells was measured via CCK‐8 assay following 4‐day culture with or without 10 µM BTKi. Statistical significance was determined by one‐way ANOVA followed by Tukey's multiple‐comparisons test. (C) Quantification of cell cycle phases (G1, S, G2/M) in wild‐type and mutant (S47G, A54G) OCI‐LY19 and Raji cells treated with or without BTKi. Data were analyzed by one‐way ANOVA. (D) Apoptosis analysis of OCI‐LY19 and Raji cells expressing wild‐type or mutant (S47G, A54G) IGLL5, treated with or without BTKi in the presence or absence of RTX. Apoptosis was assessed by flow cytometry. Statistical significance was determined by one‐way ANOVA followed by Tukey's multiple‐comparisons test. (E, F) BALB/c nude mice were subcutaneously inoculated with OCI‐LY19 (E) and Raji (F) cells expressing wild‐type, S47G‐, or A54G‐mutant IGLL5. The mice received weekly intraperitoneal injections of BTKi (10 mg/kg) or PBS (control) and were euthanized after 4 weeks. Representative images of xenograft tumors are shown. (G, H) Tumor volume (left) and tumor weights (right) of the xenografts derived from OCI‐LY19 **(G)** and Raji (H) cells. Data are presented as mean ± SD (n = 5 mice per group). Statistical significance was determined by two‐way ANOVA followed by Tukey's multiple‐comparisons test. (I, J) Representative H&E staining and immunohistochemical staining for Ki67 and cleaved Caspase3 in xenograft tumor tissues. All data are presented as the means ± SD of three biologically independent experiments. Statistical significance is denoted as ^ns^ *p* >0.05, ^*^ *p* <0.05, ^**^ *p* <0.01, ^***^ *p* <0.001.

Next, we investigated whether inhibition of the BCR pathway could attenuate the malignant phenotype associated with the IGLL5 mutation. Considering BTK as a central mediator of BCR signaling and downstream NF‐κB activation, we selected ibrutinib for further study. To determine the working concentration for subsequent experiments, we assessed BTK phosphorylation under both wild‐type and mutant conditions (IGLL5^SA7G^ and IGLL5^A54G^) and observed dose‐dependent inhibition of p‐BTK. Consistent with the cell viability IC50 of approximately 10 µM, this concentration was selected for subsequent assays (Figure 5A and Figure S5A,B). The CCK‐8 assay revealed that BTKi treatment suppressed proliferation, with a more pronounced inhibitory effect observed under mutant conditions (Figure 5B). Cell cycle analysis indicated that BTKi treatment induced arrest in both G0/G1 and G2 phases, effectively abolishing the mutation‐associated differences in cell cycle distribution (Figure 5C and Figure S5C). In parallel, apoptosis assays showed that BTKi promoted apoptosis and attenuated the anti‐apoptotic phenotype observed in mutant cells under these in vitro conditions (Figure 5D and Figure S5D). In a subcutaneous tumor model, ibrutinib significantly delayed tumor growth, with a more pronounced antitumor effect in the IGLL5^S47G^ and IGLL5^A54G^ mutant groups than in the WT group (Figure 5E–H). Consistently, mutant tumors showed increased Ki67 and reduced Caspase‐3 expression, whereas ibrutinib reversed these changes and reduced the differences between mutant and wild‐type tumors (Figure 5I,J and Figure S5E,F). Together, these data indicate that BTKi suppresses the IGLL5 mutation‐induced malignant phenotypes both in vitro and in vivo, with mutation‐dependent differences in response to ibrutinib.

### 2.6 CXCL10 and CXCL11 are Key Downstream Targets of BCR Signaling Active by IGLL5 Mutations

**FIGURE 6:**
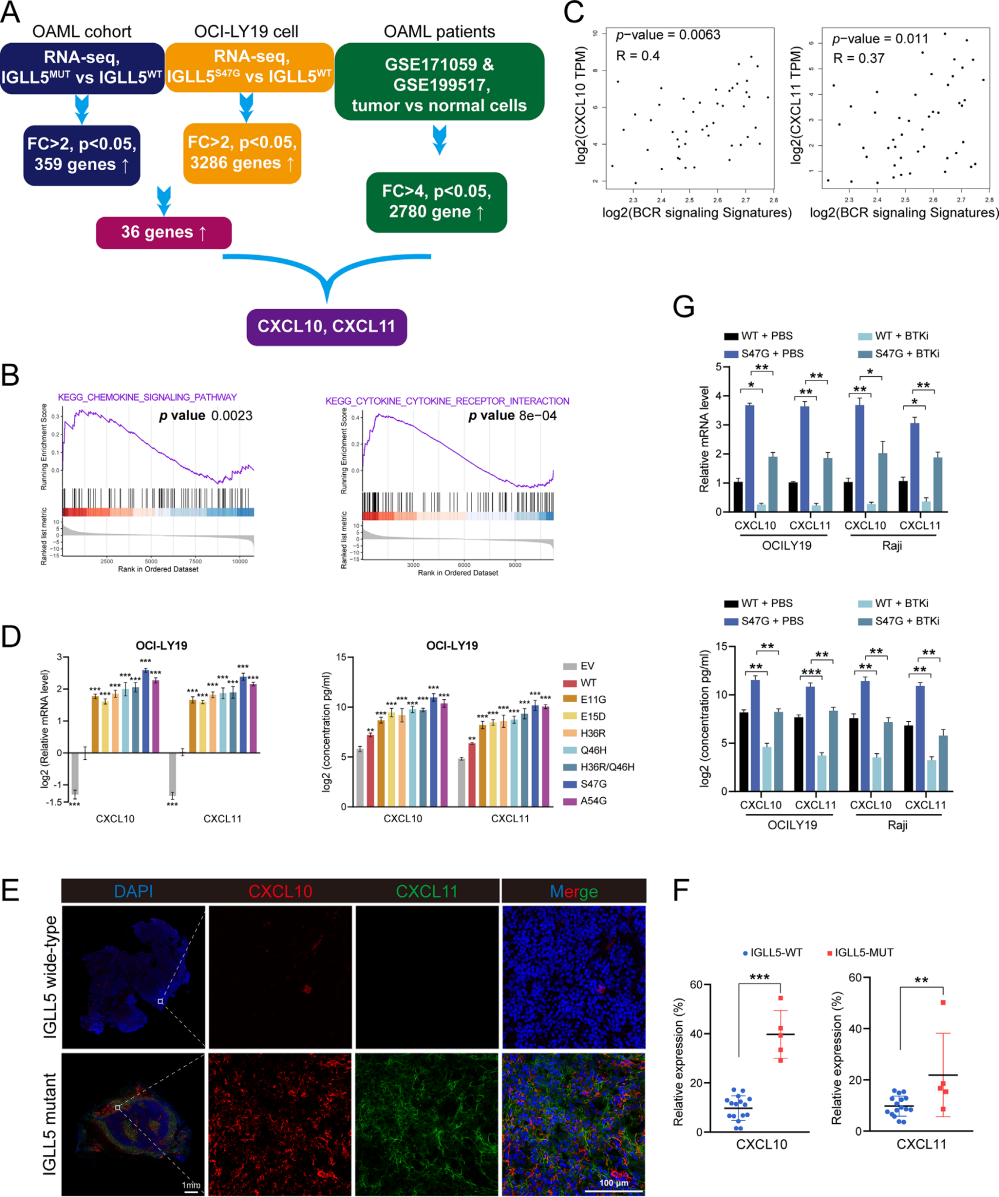
IGLL5 Mutation Leads to Upregulation of CXCL10 and CXCL11 by Activating the BCR Pathway. (A) Bioinformatics analysis identified CXCL10 and CXCL11 as oncogenic downstream targets of *IGLL5* mutants in OAML. (B) GSEA indicated that significantly enriched gene sets were associated with chemokine signaling pathway and cytokine–cytokine receptor interaction pathways in OCI‐LY19 cells transfected with IGLL5 S47G versus IGLL5 WT plasmid. (C) Correlation analysis of CXCL10 and CXCL11 expression with BCR signature in the TCGA‐DLBCL cohort. Spearman's rank correlation test was used to assess the relationship. (D) RT‐qPCR analysis and ELISA detection of CXCL10 and CXCL11 expression were conducted in Raji cell lines transduced with different IGLL5 mutants, using ACTB (*β*‐actin) as an input control. Statistical significance was determined by one‐way ANOVA followed by Tukey's multiple‐comparisons test, with significance normalized to the WT control. (E) Representative immunofluorescence images showing CXCL10(red) and CXCL11(green) staining in sections from OAML lymphoma tissues, with or without IGLL5 variants. (F) Scatter plots showing the quantified expression levels of CXCL10 and CXCL11 in immunofluorescence images from OAML lymphoma tissues. Statistical significance was determined using a two‐tailed unpaired Student's *t*‐test. (G) RT‐qPCR and ELISA analysis of CXCL10 and CXCL11 expression in Raji and OCI‐LY19 cell lines transduced with S47G or WT IGLL5, with or without ibrutinib treatment (BCR pathway inhibitor). Statistical significance was determined using one‐way ANOVA. All data are presented as the means ± SD of three biologically independent experiments. Statistical significance was determined by one‐way ANOVA followed by Tukey's multiple‐comparisons test. Statistical significance is denoted as ^*^ *p* <0.05, ^**^ *p* <0.01, ^***^ *p* <0.001.

Given that gain‐of‐function mutations potentiate BCR signal propagation, we sought to determine whether this leads to the dysregulation of downstream transcriptional networks. To explore this, we re‐analyzed the OAML cohort RNA‐seq data, revealing distinct transcriptional profiles in *IGLL5* mutant patients. Given the pronounced pro‐proliferative and anti‐apoptotic phenotypes, we generated an IGLL5^S47G^ mutant model for subsequent RNA‐seq analysis (Figure S6A). Notably, 36 genes were consistently upregulated (FoldChange > 2, *p* < 0.05) in both mutant tumor tissue and cell lines. KEGG pathway analysis indicated that these differentially expressed genes were not enriched in BCR signaling itself but were enriched in multiple downstream pathways, including NF‐*κ*B signaling, Ca^2^^+^ signaling, and Toll‐like receptor (TLR) signaling, consistent with previous reports (Figure S6B). Further integrated analysis of RNA‐seq data from two independent OAML cohorts (GSE171059, GSE199517) identified *CXCL10* and *CXCL11* as the only genes significantly overexpressed in tumor versus normal cells (Figure 6A). GSEA comparing S47G mutant and wild‐type samples revealed that S47G was associated with upregulation of the chemokine signaling pathway and cytokine‐cytokine receptor interaction pathways (Figure 6B). Moreover, investigation of the TCGA expression data revealed that both *CXCL10* and *CXCL11* positively correlated with elevated BCR signaling activity (by GSVA), supporting their roles as potential downstream effectors of the BCR in B‐cell lymphoma (Figure 6C).

*CXCL10* and *CXCL11* are associated with tumor progression in various cancers, including B‐cell malignancies [24, 25, 26]. Indeed, both chemokines were significantly upregulated in the TCGA DLBCL cohort (Figure S6C). Clinical stage further suggested that high expression levels of *CXCL10* and *CXCL11* correlated with advanced disease (Table S1 and Table S2). Moreover, survival analysis revealed that follicular lymphoma patients with high *CXCL10*/*CXCL11* expression had significantly worse outcomes than those with low expression (Figure S6D). To assess the functional impact of these mutations on expression, we conducted qPCR and ELISA assays. The results demonstrated significant upregulation of both CXCL10 and CXCL11 in IGLL5 mutant cells relative to wild‐type controls (Figure 6D and Figure S6E). Immunofluorescence analysis further showed an increased proportion of CXCL10^+^ and CXCL11^+^ lymphoma cells in IGLL5^S47G^ mutant (Figure S6F). Consistently, tissue immunofluorescence from OAML patients corroborated strong expression of CXCL10 and CXCL11 specifically in IGLL5 mutant lymphoma (Figure 6E,F). To determine whether BCR signaling mediates the upregulation of CXCL10 and CXCL11, we treated cells with ibrutinib. The BTKi reduced both mRNA and protein levels of CXCL10 and CXCL11, which were previously elevated in IGLL5^S47G^ and IGLL5^A54G^ cells (Figure 6G and Figure S6G). These results demonstrated that IGLL5 mutations drive CXCL10/CXCL11 expression via BCR pathway activation.

### 2.7 IGLL5 Mutations are Associated with CXCL10/CXCL11 Upregulation and CD8^+^ T‐Cell Recruitment and Dysfunction

**FIGURE 7:**
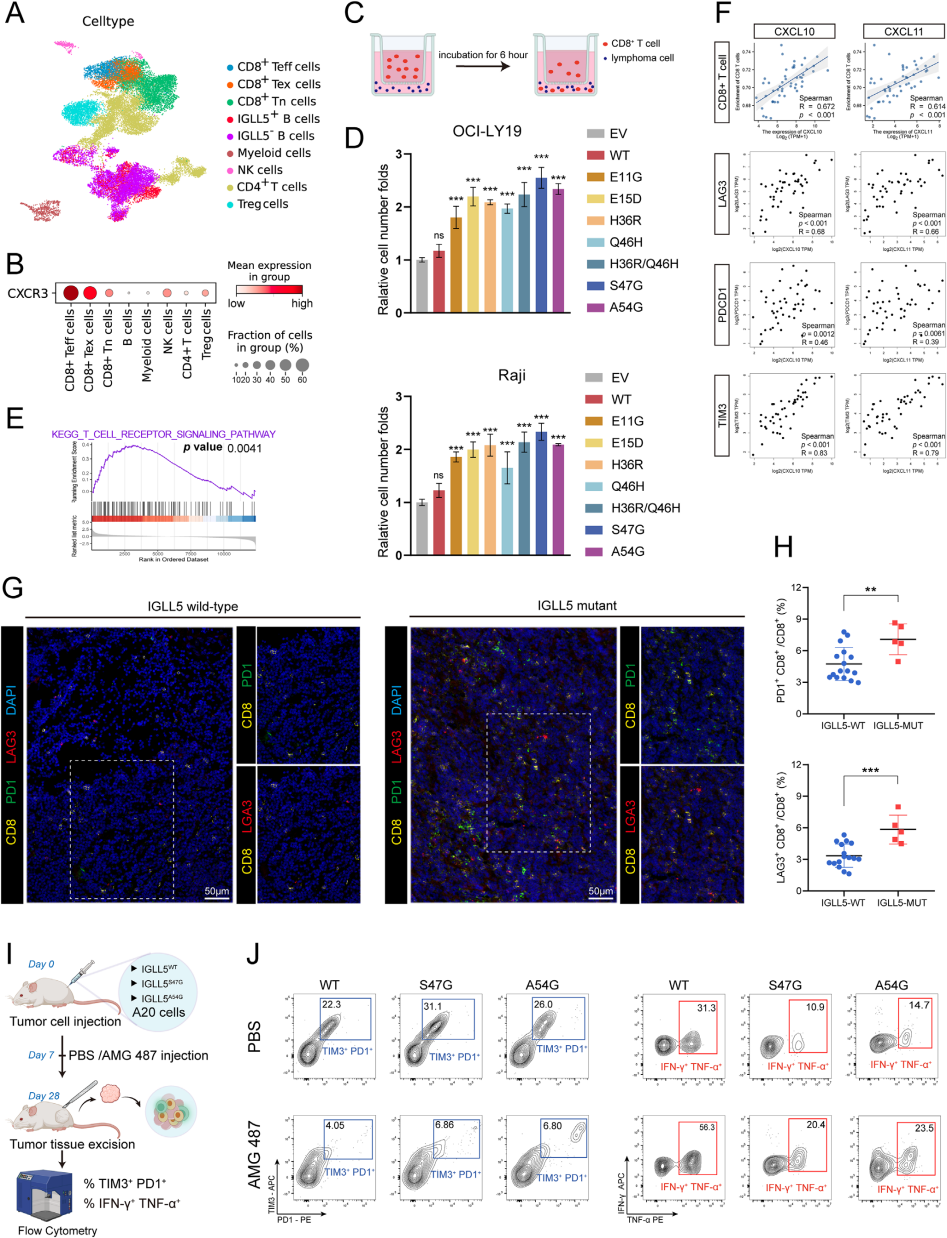
IGLL5 Mutations Promote CD8^+^ T Cell Infiltration and Exhaustion in B Lymphoma. (A) UMAP plot displaying clustering results colored by the major cellular compartments in the GSE182434 dataset. (B) *CXCR3* expression levels in the primary cellular compartments (GSE182434). (C) Schematic representation of the CD8^+^ T cell migration assay. (D) Relative migration of CD8^+^ T cells in the presence of culture supernatants from OCI‐LY19 (upper) and Raji (bottom) cell lines transduced with IGLL5 wild‐type, IGLL5 mutants, or EV as negative controls. Migration was assessed to evaluate the influence of IGLL5 variants on T cell chemotaxis, with supernatants providing insights into the immune response modulation by IGLL5 mutations. Statistical significance was determined by one‐way ANOVA followed by Tukey's multiple‐comparisons test. (E) GSEA Statistical significance was determined by one‐way ANOVA followed by Tukey's multiple‐comparisons test. (F) Correlation analysis of *CXCL10* and *CXCL11* expression with CD8^+^ T cell signature and markers of exhausted CD8^+^ T cells (*PD‐1*, *LAG‐3*, *TIM‐3*) in the TCGA‐DLBCL cohort. Spearman correlation analysis was performed to assess the relationship. (G) Representative immunofluorescence images of CD8 (yellow), LAG3 (red), and PD1 (green) in OAML tissues with or without IGLL5 mutations. (H) Scatter plots showing the percentages of LAG3^+^CD8^+^ (upper), PD1^+^CD8^+^ (bottom) T cells among total CD8^+^ T cells. Statistical significance was determined using two‐tailed unpaired Student's *t*‐test. (I) Schematic illustration of the in vivo experimental design. BALB/c mice were subcutaneously inoculated with A20 cells expressing WT or mutant (MUT) IGLL5. One week after tumor inoculation, mice received AMG 487 (a CXCR3 antagonist that inhibits the CXCL10/CXCL11–CXCR3 axis) or PBS as a control. After 4 weeks, tumors were harvested, dissociated into single‐cell suspensions, and analyzed by flow cytometry. (J) Representative flow cytometry plots showing PD‐1^+^TIM‐3^+^CD8^+^ T‐cells and INFγ^+^TNFα^+^CD8^+^ T‐cells in the subcutaneous tumor model. Quantification across experimental groups is shown in Figure S7E. Representative plots are shown from independent mice. Statistical significance is denoted as ^ns^ *p* >0.05, ^**^ *p* <0.01, ^***^ *p* <0.001.

As chemokines, CXCL10 and CXCL11 are known to critically regulate immune cell recruitment, migration, and activation. To identify immune cell populations interacting with CXCL10^+^/CXCL11^+^ B lymphoma cells, we analyzed single‐cell RNA‐seq data (GSE182434) to characterize the transcriptional profiles of both chemokine‐expressing B cells and their potential interacting partners (Figure 7A). However, due to the low abundance of CXCL10‐ or CXCL11‐expressing B cells, we were unable to reliably infer direct cell‐cell communication probabilities using the “CellChat” package. Instead, we examined the expression of CXCR3, the common receptor for both ligands. As shown in Figure 7B, CXCR3 was markedly enriched in CD8^+^ T cells, particularly effector (Teff) and exhausted (Tex) subsets. We further used “CellChat” to compare interaction potentials between IGLL5^+^ and IGLL5^−^ B cells and various immune subgroups (Figure S7A). Interestingly, no significant differences in inferred interactions were observed, suggesting that IGLL5 expression alone may not substantially influence T‐cell chemotaxis or immune communications (Figure S7B).

To determine whether IGLL5‐mutant lymphoma cells enhance CD8^+^ T cell infiltration through CXCL10 and CXCL11 upregulation, we conducted a CD8^+^ T cell migration assay. Results revealed that IGLL5 mutation significantly promoted CD8^+^ T cell migration compared to wild‐type controls in OCI‐LY19 and Raji cells (Figure 7C,D) [27], indicating that mutant IGLL5 facilitates CD8^+^ T cell recruitment via increased chemokine secretion. Although mutant IGLL5 lymphoma cells recruit both Teff and Tex CD8^+^ T cells, these subsets exhibit divergent functional states. This Tex phenotype aligns with T cell exhaustion documented in various cancers [28, 29, 30]. CXCL10 and CXCL11 have been implicated in cancer progression in OAML. Based on these observations, we hypothesized that CXCL10 and CXCL11 may contribute to CD8+ T‐cell dysfunction by promoting their accumulation within the tumor microenvironment. Furthermore, persistent TCR activation, which was enriched in IGLL5^S47G^ cells compared to wild‐type controls (Figure 7E), may cooperate with chemokine‐associated recruitment to promote an exhaustion‐associated phenotype rather than directly establish T‐cell exhaustion [31]. Analysis of TCGA data confirmed a positive correlation between exhaustion markers and expression levels of both *CXCL10* and *CXCL11* (Figure 7F). Multicolor immunofluorescence staining revealed that IGLL5 wild‐type tumors exhibited low CXCL10/CXCL11 expression and limited CD8^+^ T cell infiltration. In contrast, IGLL5 mutant tumors displayed abundant CD8^+^ T cell infiltration accompanied by an exhausted phenotype characterized by high PD‐1 and LAG‐3 expression (Figure 7G,H). Finally, using an A20 subcutaneous tumor model and multicolor flow cytometry, we found that IGLL5 mutation significantly increased the proportion of PD‐1^+^ TIM‐3^+^ CD8^+^ T cells among tumor‐infiltrating lymphocytes, whereas pharmacologic interruption of the CXCL10/CXCL11‐CXCR3 axis with AMG 487, a CXCR3 antagonist, partially reversed this phenotype (Figure 7I and Figure S7C–E). Consistently, CD8^+^ T cells exposed to IGLL5‐mutant lymphoma cells showed reduced IFN‐γ and TNF‐α production, which was partially restored by AMG 487 treatment (Figure 7J). These data support a role for the CXCL10/CXCL11‐CXCR3 axis in IGLL5 mutation‐associated CD8^+^ T‐cell dysfunction. Together, these findings support a model in which *IGLL5* mutation is associated with CXCL10/CXCL11 upregulation and CXCR3‐dependent CD8^+^ T‐cell recruitment and dysfunction, thereby contributing to an exhaustion‐associated immune microenvironment.

### 2.8 BTKi and RTX Combination Treatment Showed Better Efficacy in IGLL5‐Mutant B‐Cell Lymphoma

**FIGURE 8:**
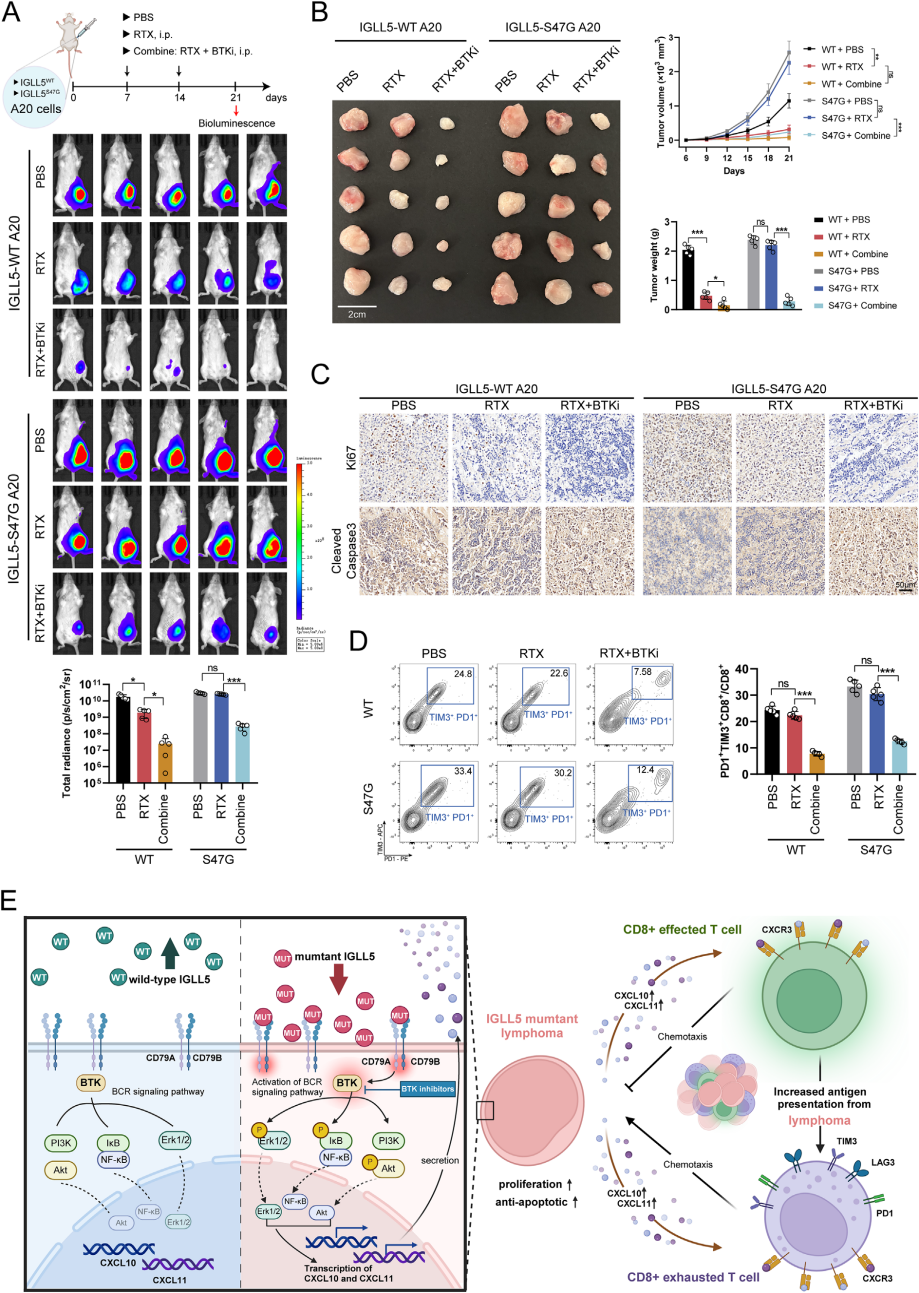
BTKi Combined With RTX Showed Superior Efficacy Against IGLL5‐mutant B‐cell Lymphoma. (A) Schematic of the in vivo experimental design. BALB/c mice were subcutaneously inoculated with A20 cells transfected with WT or S47G IGLL5 plasmids and randomized into three groups receiving weekly intraperitoneal injections of PBS, RTX alone, or RTX combined with BTKi. Tumor progression was monitored by in vivo bioluminescence imaging on day 21 (top). Representative in vivo bioluminescence images (middle) and quantification of total radiance (bottom) of subcutaneous xenografts are shown. Data are presented as mean ± SD (n = 5 mice per group). Statistical significance was determined by one‐way ANOVA followed by Tukey's multiple‐comparisons test. (B) Representative images of subcutaneous xenografts (left), tumor volume measurements (top right), and tumor weights (bottom right). Statistical significance was determined by two‐way ANOVA followed by Tukey's multiple‐comparisons test. (C) Representative IHC staining for Ki67 and Cleaved Caspase3 in xenograft tumor tissues. (D) Flow cytometry plots (left) demonstrating exhausted CD8^+^ T cells in the subcutaneous tumor model, with the corresponding bar graph (right) depicting their proportion relative to total CD8^+^ T cells across the indicated experimental groups. Statistical significance was determined by one‐way ANOVA followed by Tukey's multiple‐comparisons test. (E) Schematic model illustrating that selected IGLL5 mutations enhance BCR‐related signaling through association with the CD79A/CD79B complex and are associated with CXCL10/CXCL11 upregulation, increased CD8^+^ T‐cell infiltration, and an exhaustion‐associated immune microenvironment, thereby contributing to lymphoma progression. All data are presented as the means ± SD of three biologically independent experiments. Statistical significance is denoted as ^ns^ *p* >0.05, ^*^ *p* <0.05, ^***^ *p* <0.001.

To compare the effects of RTX monotherapy and its combination with BTK inhibition in an IGLL5‐mutant context, we evaluated this strategy in an A20 model expressing the IGLL5^S47G^ mutation. Subsequent in vivo, combined treatment with RTX and BTKi suppressed tumor growth more effectively than RTX alone and was accompanied by increased tumor cell apoptosis (Figure 8A–C and Figure S8A,B). In addition, RTX monotherapy did not substantially reduce the proportion of exhaustion‐associated CD8^+^ T cells in either the wild‐type or S47G mutant tumor microenvironment, whereas the combination treatment decreased this phenotype in the S47G model (Figure 8D). These findings indicate that the addition of BTK inhibition enhances the antitumor effect observed with RTX alone in this experimental setting and is consistent with suppression of IGLL5‐associated BCR signaling in vivo.

## 3 Discussion

In this study, we performed transcriptome profiling of the OAML cohort and, for the first time, identified molecular signatures associated with IGLL5 mutations. Through functional and mechanistic investigation, we found that S47G and A54G IGLL5 mutants enhance association with the CD79A/CD79B complex and are accompanied by activation of BCR‐related signaling. This signaling is associated with upregulation of CXCL10 and CXCL11, increased CD8^+^ T‐cell infiltration, and an exhaustion‐associated dysfunctional phenotype (Figure 8E).

Our findings support an association between selected *IGLL5* mutations and malignant progression‐associated phenotypes in OAML, although the functional consequences vary across mutations. Among the seven variants, S47G exhibited the strongest oncogenic potential and showed the most pronounced difference in direct in vitro response to RTX, thereby suggesting a potential role in modulating drug sensitivity or microenvironmental crosstalk [32, 33]. It should also be noted that the effects of RTX observed in our in vitro assays reflect only direct cellular responses and do not capture its dominant immune‐mediated mechanisms in vivo, particularly Antibody‐Dependent Cellular Cytotoxicity and Complement‐Dependent Cytotoxicity. Accordingly, these data should be interpreted as an altered direct in vitro response rather than clinically meaningful rituximab resistance. Conversely, A54G, Q46H, and the compound variant H36R+Q46H conferred moderate effects, while E11G, E15D, and H36R exhibited only mild phenotypes. *IGLL5* is an immunoglobulin lambda‐like gene located within the immunoglobulin lambda locus and is expressed in B‐cell lineages, but its physiological role in normal B cells remains incompletely understood [34]. Although IGLL5 belongs to the surrogate light‐chain family, its function is not as well defined as that of the classical surrogate light‐chain components involved in early B‐cell development [35]. In this context, our data suggest that wild‐type IGLL5 can associate with BCR‐related signaling, whereas specific mutations further potentiate this activity and confer aberrant pro‐tumor effects.

Although enhanced interaction with CD79A/B appears to be an important mechanism underlying this process, it is unlikely to be the sole mechanism. CD79A and CD79B are well‐established signaling subunits of the BCR complex and are required for BCR surface expression and downstream signaling activation. In line with this, our additional experiments showed that knockdown of CD79A and/or CD79B attenuated BCR‐related signaling in IGLL5‐mutant cells, supporting functional dependency on CD79A/B. However, these mechanistic observations were derived primarily from an endogenous IGLL5 knockout background followed by re‐expression of FLAG‐tagged WT or mutant IGLL5, which, although suitable for controlled comparison of mutation‐specific effects, may not fully recapitulate endogenous protein expression and regulation. Therefore, the proposed IGLL5‐CD79A/B‐BCR signaling axis should be interpreted as a supported mechanistic model rather than a definitively established endogenous mechanism. Given that the molecular function and interaction landscape of IGLL5 remain incompletely defined, additional binding partners or parallel pathways may also contribute to the mutant phenotype. Consistently, analysis of the TCGA cohort showed that IGLL5 was elevated in DLBCL, supporting the notion that IGLL5 may participate in B‐cell signaling even in the absence of mutation.

Structurally, the mutations clustered near the N‐terminal signaling domain of IGLL5, a region analogous to immunoglobulin light chains and likely essential for molecular recognition. Mutations such as S47G, A54G, and H36R may enhance the flexibility of this domain, thereby increasing binding affinity and specificity. This observation aligns with the recognized importance of structural plasticity in biomolecular interactions [36]. Notably, S47 is highly conserved, and its substitution to glycine likely induces significant conformational alterations, explaining the strong impact of S47G on BCR activation. The activation of BCR signaling by IGLL5 mutations not only promotes malignant B‐cell proliferation but also shapes therapeutic response. Among the downstream pathways, PI3K/Akt signaling enhances resistance to apoptosis, providing a survival advantage; Erk signaling facilitates rapid cell cycle progression; while activation of the NF‐κB pathway primarily drives tumor growth by inducing transcription of pro‐inflammatory and chemotactic factors, including CXCL10 and CXCL11 [26, 37]. Collectively, these signaling events enable malignant B cells to proliferate and persist within a tumor‐supportive microenvironment.

The tumor microenvironment is a critical determinant of lymphoma progression. CXCL10 and CXCL11 are markedly overexpressed in several B‐cell malignancies [25, 38], where they modulate the tumor immune milieu. Initially, these chemokines recruit CD8^+^ T cells to the tumor site and may elicit an anti‐tumor immune response. In our study, the IGLL5 mutation was associated with increased expression of CXCL10 and CXCL11 and enhanced CD8^+^ T cell infiltration. Since CXCL10/CXCL11 are established ligands for CXCR3, these findings support a model in which IGLL5 mutant‐driven BCR signaling reshapes the immune microenvironment through a mechanism involving the CXCL10/CXCL11‐CXCR3 axis. Importantly, pharmacologic interruption of this pathway with AMG 487 partially reversed the increase in PD‐1^+^ TIM‐3^+^ CD8^+^ T cells and partially restored IFN‐γ and TNF‐α production, providing functional support for the involvement of this chemokine axis in CD8^+^ T cell dysfunction. However, the transition from chemokine‐mediated recruitment to full establishment of T‐cell exhaustion is likely multifactorial and cannot be attributed to CXCL10/CXCL11‐CXCR3 signaling alone. Therefore, our data suggest that activation of the CXCL10/CXCL11‐CXCR3 axis may be one potential mechanism by which IGLL5 mutations shape an exhaustion associated immune microenvironment in OAML [39, 40]. Beyond immune regulation, BCR signaling may also influence tumor–stroma interactions, further contributing to lymphoma progression [41, 42]. Together, these findings suggest that IGLL5 mutations promote lymphoma progression through coordinated effects on tumor‐intrinsic BCR signaling and the surrounding immune microenvironment.

Our findings align with previous reports that MALT lymphomas frequently harbor alterations in chromatin remodeling, BCR/NF‐κB, and NOTCH signaling pathways. Nevertheless, genetic lesions differ substantially across anatomical sites, reflecting site‐specific pathogenic mechanisms [43]. Concurrent *IGLL5* and *TNFAIP3* mutations were identified in two cases in our cohort; however, *TNFAIP3* mutations did not co‐occur with the S47G or A54G IGLL5 variants. Given the limited cohort size, the significance of this mutational combination remains unclear. As *TNFAIP3* is a key negative regulator of NF‐κB signaling, this finding may indicate a distinct biological context in a subset of OAML and warrants further study in larger cohorts. The high prevalence of IGLL5 mutations in OAML further supports this unique genetic profile compared with other MALT lymphomas. However, given that IGLL5 mutations were identified in only a subset of cases in our cohort, they should be considered a recurrent molecular feature rather than a diagnostic marker of OAML. Validation in independent cohorts and across other orbital lymphoproliferative conditions will be required to define their specificity and potential clinical utility. Moreover, the similarly elevated mutation frequency of IGLL5 in OAML and VRL suggests potential biological and clinical parallels between these entities, both arising in ocular or periocular tissues. These observations underscore the importance of site‐specific studies to dissect the interplay between genetic alterations and microenvironmental influences in lymphoma progression. Such insights may inform therapeutic strategies.

Our study also highlights the context‐dependent nature of IGLL5 mutations. For instance, IGLL5 mutations in CLL have been associated with reduced expression levels and a favorable prognosis [14], whereas in DLBCL they correlated with poorer prognoses, reflecting the diverse roles across malignancies. Such variability may arise from mutation‐specific effects on transcriptional regulation. In OAML, we observed that IGLL5 mutations markedly enhanced BCR signaling, supporting a gain‐of‐function mechanism. This divergence emphasizes the importance of mutation‐specific studies to delineate how IGLL5 contributes to disease biology in distinct lymphoma subtypes. Importantly, our in vivo data showed that adding BTKi to RTX achieved greater antitumor activity than RTX monotherapy in the IGLL5‐mutant model. This finding supports combined targeting of BCR signaling and CD20 as a mechanism‐based therapeutic strategy that warrants further evaluation in IGLL5‐mutant OAML. Although radiotherapy remains a major standard treatment for localized OAML, and clear clinical synergy between ibrutinib and rituximab has not yet been established, our results provide a rationale for further evaluation of this combination. The translational relevance of this combination in OAML still requires further clinical and mechanistic evidence.

Several limitations should be considered. Mechanistic studies were conducted primarily in systemic lymphoma cell lines [22, 23, 44], which may not fully recapitulate the site‐specific biology of OAML. Although the working concentration of ibrutinib was selected based on dose‐dependent p‐BTK inhibition, potential off‐target effects at higher concentrations cannot be fully excluded. Functional analyses were limited to the S47G and A54G IGLL5 mutants and therefore reflect mutation‐associated effects in a subset of cases. Although AMG 487 supports involvement of the CXCL10/CXCL11–CXCR3 axis, ligand‐specific validation is lacking, and the causal link between CD8^+^ T‐cell recruitment and the full establishment of T‐cell exhaustion remains incompletely defined. Enhanced interaction with CD79A/B may not represent the sole mechanism, as additional interacting partners or parallel pathways cannot be excluded. Finally, the relatively small cohort size precludes robust assessment of infrequent co‐mutation events, including concurrent TNFAIP3 alterations.

In conclusion, our findings support a mutation‐associated mechanism in a subset of OAML, in which selected IGLL5 mutants enhance BCR‐related signaling and are associated with CXCL10/CXCL11 upregulation, increased CD8^+^ T‐cell infiltration, and an exhaustion‐associated immune microenvironment. Importantly, combined inhibition with BTKi and RTX suppressed sustained BCR‐related signaling in our experimental models, supporting a mechanism‐based therapeutic strategy that warrants further validation. These findings deepen our understanding of OAML pathogenesis and provide a rationale for therapeutic approaches targeting this signaling–immune axis.

## 4 Experimental Section

### 4.1 Patient Cohort

The study was approved by the Human Ethics Committee of the Hospital (NO. 2022‐SRFA‐334), and each patient signed informed consent before the research started. Transcription data from patient samples (n = 21) were obtained from The First Affiliated Hospital with Nanjing Medical University, as described previously [6]. These specimens were collected from surgically removed tumors prior to any treatment and were directly frozen in liquid nitrogen. A portion of each sample underwent histopathological analysis, while the remainder was used for DNA extraction to identify *IGLL5* mutations. Diagnosis was confirmed based on standard histological criteria and immunohistochemistry, including markers such as CD20, CD79a, CD3, and CD10. In cases where immunophenotyping alone was inconclusive, genetic analysis was performed to rule out other lymphoproliferative disorders.

### 4.2 Cell Lines and Cell Culture

Human Non‐Hodgkin Lymphoma cell lines OCI‐LY19 and Raji, with murine lymphoma cell line A20, were obtained from the Cell Bank (Chinese Academy of Sciences). OCI‐LY19 cells were maintained in MEM (Thermo Fisher Scientific, USA) supplemented with 20% fetal bovine serum (FBS; Gibco, USA) and 1% penicillin/streptomycin (Beyotime Biotechnology, China). A20 and Raji cells were cultured in RPMI‐1640 (Thermo Fisher Scientific, USA) containing 10% FBS and 1% penicillin/streptomycin. All cell lines were maintained in a humidified incubator with 5% CO_2_ at 37°C. Regular testing for mycoplasma contamination was performed using RT‐qPCR to ensure cell culture quality.

### 4.3 Cell Transfection and Transduction

Three individual lentiviral vectors containing shRNAs targeting human *IGLL5* were purchased from Tsingke Biotechnology (Shanghai, China) to knock down endogenous *IGLL5* expression. A non‐targeting shRNA was used as the negative control. The wild‐type full‐length coding sequence of IGLL5 in pLex‐MCS, as well as various IGLL5 mutants (E11G, E15D, H36R, Q46H, S47G, A54G, and the H36R+Q46H compound mutant), were synthesized by Jikai Gene Chemical Technology Co. Ltd (Nanjing, China). All constructs were engineered with a C‐terminal FLAG tag (DYKDDDDK) for analysis of IGLL5 cellular localization, co‐immunoprecipitation (Co‐IP), and Western blot. For transfection, Lipofectamine 3000 (Invitrogen, CA, USA) was used according to the manufacturer's protocol to introduce the shRNAs and plasmid vectors into the cells. The efficiency of IGLL5 silencing was verified by qRT‐PCR and Western blot. For wild‐type and mutant IGLL5 constructs, Sanger sequencing was used to confirm successful transfection.

### 4.4 RNA Isolation, RNA Sequencing (RNAseq) and Quantitative Real‐Time PCR (qRT‐PCR)

For RNA isolation, RNA was extracted from OAML tissue specimens and cell lines using TRIzol reagent (Invitrogen, USA) according to the manufacturer's instructions. For the RNA sequence, Poly‐A mRNA was extracted from total RNA using poly‐T oligo magnetic beads and subsequently reverse transcribed into first‐strand cDNA with random hexamers. Second‐strand cDNA synthesis was performed using DNA polymerase I and RNase H. PCR was used to enrich adapter‐linked DNA fragments for library preparation. The library quality was assessed with an Agilent 2100 bioanalyzer, and concentrations were measured by Qubit and qPCR. Sequencing was performed on the Illumina Novaseq 6000 platform, and reads were aligned to the hg38 genome.

For qRT‐PCR, RNA was extracted as described above. cDNA was synthesized from total RNA using HiScript RT Mix (Vazyme, China). qRT‐PCR was performed to quantify relative RNA levels using the SYBR Green PCR kit (Vazyme, China) on a Q7 Real‐Time PCR System (Applied Biosystems, USA). The comparative CT (2^−ΔΔCT^) method was used to calculate gene expression, with *β*‐actin as the internal control. Primers used for this analysis are listed in Table S3.

### 4.5 Immunoblotting

Proteins were isolated from cells using RIPA lysis buffer supplemented with protease inhibitors (Beyotime Biotechnology, China). After extraction, protein samples were separated on SDS‐PAGE gels and subsequently transferred onto PVDF membranes (Solarbio, China). The membranes were blocked with QuickBlock solution (Beyotime Biotechnology, China) and then incubated overnight at 4°C with primary antibodies, with anti‐*β*‐actin used as a loading control for normalization. Following primary antibody incubation, the membranes were treated with HRP‐conjugated secondary antibodies for 2 h at room temperature. Protein bands were visualized using an electrochemiluminescence (ECL) detection system (YEASEN, Shanghai, China). Details of the antibodies utilized in this experiment can be found in Table S4.

### 4.6 Statistical Analysis

Statistical analysis and data visualization were performed using R software (version 4.1.3) and GraphPad Prism 8.0 (GraphPad, San Diego, CA, USA). Quantitative data are presented as mean ± SD unless otherwise indicated. Comparisons between two groups were performed using a two‐tailed unpaired Student's *t*‐test. For comparisons involving three or more groups, one‐way or two‐way analysis of variance (ANOVA) was used as appropriate, followed by Tukey's multiple‐comparisons post hoc test for pairwise group comparisons. Where multiple comparisons were performed, *p* values were adjusted accordingly using the post hoc test implemented in the corresponding analysis. For survival analysis, the Kaplan–Meier method was used to estimate survival distributions, and differences between groups were assessed using the log‐rank test. Correlation analyses were performed using Spearman's rank correlation test. All statistical tests were two‐sided, and *p* < 0.05 was considered statistically significant.

More details regarding the methods can be found in the Supporting Information.

## Author Contributions

CXJ and LH conceived of the study and carried out its design. ZAD performed the experiments. ZAD, WHY, and ZCY conducted the statistical analysis. SXP, LHY, DSY, WZJ, QS, QG, ZH, and SSY collected clinical samples. ZAD wrote the paper and CXJ revised the paper. All authors read and approved the final manuscript.

## Conflicts of Interest

The authors declare no conflicts of interest.

## Supporting information




**Supporting File 1**: advs75628‐sup‐0001‐SuppMat.docx.

## Data Availability

The data that support the findings of this study are available on request from the corresponding author. The data are not publicly available due to privacy or ethical restrictions.

## References

[1] J. A. Ferry , C. Y. Fung , L. Zukerberg , et al., “Lymphoma of the Ocular Adnexa: a Study of 353 Cases,” The American Journal of Surgical Pathology 31, no. 2 (2007): 170–184, 10.1097/01.pas.0000213350.49767.46. 17255761

[2] Y. Liang , R. Y. Fu , X. L. Liu , et al., “Long‐Term Survival Outcomes of Patients with Primary Ocular Adnexal MALT Lymphoma: a Large Single‐Center Cohort Study,” Cancer Medicine 12, no. 3 (2023): 2514–2523, 10.1002/cam4.5092. 35906828 PMC9939090

[3] A. J. M. Ferreri , R. Dolcetti , M.‐Q. Du , et al., “Ocular Adnexal MALT Lymphoma: an Intriguing Model for Antigen‐driven Lymphomagenesis and Microbial‐targeted Therapy,” Annals of Oncology 19, no. 5 (2008): 835–846, 10.1093/annonc/mdm513. 17986622

[4] M. Raderer , B. Kiesewetter , and A. J. Ferreri , “Clinicopathologic Characteristics and Treatment of Marginal Zone Lymphoma of Mucosa‐associated Lymphoid Tissue (MALT lymphoma),” CA: A Cancer Journal for Clinicians 66, no. 2 (2016): 153–171. 26773441 10.3322/caac.21330

[5] E. Zucca and F. Bertoni , “The Spectrum of MALT Lymphoma at Different Sites: Biological and Therapeutic Relevance,” Blood 127, no. 17 (2016): 2082–2092, 10.1182/blood-2015-12-624304. 26989205

[6] A. Zhao , F. Wu , Y. Wang , J. Li , W. Xu , and H. Liu , “Analysis of Genetic Alterations in Ocular Adnexal Mucosa‐Associated Lymphoid Tissue Lymphoma with Whole‐Exome Sequencing,” Frontiers in Oncology 12 (2022): 817635, 10.3389/fonc.2022.817635. 35359413 PMC8962736

[7] J. J. Kwak , K. S. Lee , J. Lee , et al., “Next‐Generation Sequencing of Vitreoretinal Lymphoma by Vitreous Liquid Biopsy: Diagnostic Potential and Genotype/Phenotype Correlation,” Investigative Opthalmology & Visual Science 64, no. 14 (2023): 27, 10.1167/iovs.64.14.27. PMC10664732 37975847

[8] K. Yoshifuji , D. Sadato , T. Toya , et al., “Impact of Genetic Alterations on central Nervous System Progression of Primary Vitreoretinal Lymphoma,” Haematologica 109, no. 11 (2024): 3641–3649. 38841798 10.3324/haematol.2023.284953 PMC11532695

[9] I. Bonzheim , P. Sander , J. Salmeron‐Villalobos , et al., “The Molecular Hallmarks of Primary and Secondary Vitreoretinal Lymphoma,” Blood Advances 6, no. 5 (2022): 1598–1607. 34448823 10.1182/bloodadvances.2021004212 PMC8905692

[10] H. Zou , W. Liu , X. Wang , et al., “Dynamic Monitoring of Circulating Tumor DNA Reveals Outcomes and Genomic Alterations in Patients with Relapsed or Refractory Large B‐cell Lymphoma Undergoing CAR T‐cell Therapy,” Journal for ImmunoTherapy of Cancer 12, no. 3 (2024): 008450, 10.1136/jitc-2023-008450. PMC11146396 38443094

[11] S.‐S. Li , X.‐H. Zhai , H.‐L. Liu , et al., “Whole‐exome Sequencing Analysis Identifies Distinct Mutational Profile and Novel Prognostic Biomarkers in Primary Gastrointestinal Diffuse Large B‐cell Lymphoma,” Experimental Hematology & Oncology 11, no. 1 (2022): 71, 10.1186/s40164-022-00325-7. 36243813 PMC9569083

[12] R. I. Panea , C. L. Love , J. R. Shingleton , et al., “The Whole‐genome Landscape of Burkitt Lymphoma Subtypes,” Blood 134, no. 19 (2019): 1598–1607, 10.1182/blood.2019001880. 31558468 PMC6871305

[13] M. D'Agostino , G. M. Zaccaria , B. Ziccheddu , et al., “Early Relapse Risk in Patients with Newly Diagnosed Multiple Myeloma Characterized by Next‐generation Sequencing,” Clinical Cancer Research 26, no. 18 (2020): 4832–4841, 10.1158/1078-0432.CCR-20-0951. 32616499

[14] C. Pérez‐Carretero , M. Hernández‐Sánchez , T. González , et al., “Chronic Lymphocytic Leukemia Patients with IGH Translocations Are Characterized by a Distinct Genetic Landscape with Prognostic Implications,” International Journal of Cancer 147, no. 10 (2020): 2780–2792, 10.1002/ijc.33235. 32720348

[15] D. A. Russler‐Germain , K. Krysiak , C. Ramirez , et al., “Mutations Associated with Progression in Follicular Lymphoma Predict Inferior Outcomes at Diagnosis: Alliance A151303,” Blood Advances 7, no. 18 (2023): 5524–5539, 10.1182/bloodadvances.2023010779. 37493986 PMC10514406

[16] S. Kalmbach , M. Grau , M. Zapukhlyak , et al., “Novel Insights into the Pathogenesis of Follicular Lymphoma by Molecular Profiling of Localized and Systemic Disease Forms,” Leukemia 37, no. 10 (2023): 2058–2065, 10.1038/s41375-023-01995-w. 37563306 PMC10539171

[17] S. M. Setayesh , L. J. Ndacayisaba , K. E. Rappard , et al., “Targeted Single‐cell Proteomic Analysis Identifies New Liquid Biopsy Biomarkers Associated with Multiple Myeloma,” npj Precision Oncology 7, no. 1 (2023): 95, 10.1038/s41698-023-00446-0. 37723227 PMC10507120

[18] H. Hosoi , S. Tabata , H. Kosako , et al., “IGLL5 controlled by Super‐enhancer Affects Cell Survival and MYC Expression in Mature B‐cell Lymphoma,” Leuk Res Rep 21 (2024): 100451. 38444524 10.1016/j.lrr.2024.100451 PMC10912717

[19] P. Milpied , I. Cervera‐Marzal , M. L. Mollichella , et al., “Human Germinal Center Transcriptional Programs Are De‐synchronized in B Cell Lymphoma,” Nature Immunology 19, no. 9 (2018): 1013–1024, 10.1038/s41590-018-0181-4. 30104629

[20] J. M. Irish , D. K. Czerwinski , G. P. Nolan , and R. Levy , “Altered B‐cell Receptor Signaling Kinetics Distinguish human Follicular Lymphoma B Cells from Tumor‐infiltrating Nonmalignant B Cells,” Blood 108, no. 9 (2006): 3135–3142, 10.1182/blood-2006-02-003921. 16835385 PMC1895530

[21] J. M. Andrews , S. C. Pyfrom , J. A. Schmidt , et al., “Loss of Synergistic Transcriptional Feedback Loops Drives Diverse B‐cell Cancers,” EBioMedicine 71 (2021): 103559, 10.1016/j.ebiom.2021.103559. 34461601 PMC8403728

[22] B. Korona , D. Korona , W. Zhao , A. C. Wotherspoon , and M. Q. Du , “GPR34 activation Potentially Bridges Lymphoepithelial Lesions to Genesis of Salivary Gland MALT Lymphoma,” Blood 139, no. 14 (2022): 2186–2197, 10.1182/blood.2020010495. 34086889

[23] J. Shi , T. Zhu , H. Lin , et al., “Proteotranscriptomics of Ocular Adnexal B‐cell Lymphoma Reveals an Oncogenic Role of Alternative Splicing and Identifies a Diagnostic Marker,” Journal of Experimental & Clinical Cancer Research 41, no. 1 (2022): 234, 10.1186/s13046-022-02445-8. 35906682 PMC9338531

[24] T. H. Schreiber , V. V. Deyev , J. D. Rosenblatt , and E. R. Podack , “Tumor‐induced Suppression of CTL Expansion and Subjugation by gp96‐Ig Vaccination,” Cancer Research 69, no. 5 (2009): 2026–2033, 10.1158/0008-5472.CAN-08-3706. 19223534 PMC2676230

[25] X. Zhou , S. Guo , and Y. Shi , “Comprehensive Analysis of the Expression and Significance of CXCLs in human Diffuse Large B‐cell Lymphoma,” Scientific Reports 12, no. 1 (2022): 2817, 10.1038/s41598-022-06877-2. 35181719 PMC8857324

[26] K. Wenzl , M. K. Manske , V. Sarangi , et al., “Loss of TNFAIP3 Enhances MYD88L265P‐driven Signaling in non‐Hodgkin Lymphoma,” Blood Cancer Journal 8, no. 10 (2018): 97, 10.1038/s41408-018-0130-3. 30301877 PMC6177394

[27] H. Li , J. Chen , Z. Li , et al., “S100A5 Attenuates Efficiency of Anti‐PD‐L1/PD‐1 Immunotherapy by Inhibiting CD8 + T Cell‐Mediated Anti‐Cancer Immunity in Bladder Carcinoma,” Advanced Science 10, no. 25 (2023): 2300110, 10.1002/advs.202300110. 37414584 PMC10477882

[28] C. Schütz , S. Inselmann , S. Saussele , et al., “Expression of the CTLA‐4 Ligand CD86 on Plasmacytoid Dendritic Cells (pDC) Predicts Risk of Disease Recurrence after Treatment Discontinuation in CML,” Leukemia 32, no. 4 (2018): 1054. 29381150 10.1038/leu.2017.348

[29] A. Rej , A. Paladhi , S. Daripa , et al., “Galunisertib Synergistically Potentiates the Doxorubicin‐mediated Antitumor Effect and Kickstarts the Immune System against Aggressive Lymphoma,” International Immunopharmacology 114 (2023): 109521, 10.1016/j.intimp.2022.109521. 36470118

[30] L. Li , M. Zhao , C. H. Kiernan , et al., “Ibrutinib Directly Reduces CD8+T Cell Exhaustion Independent of BTK,” Frontiers in Immunology 14 (2023): 1201415, 10.3389/fimmu.2023.1201415. 37771591 PMC10523025

[31] A. Baessler and D. A. A. Vignali , “T Cell Exhaustion,” Annual Review of Immunology 42, no. 1 (2024): 179–206, 10.1146/annurev-immunol-090222-110914. 38166256

[32] A. Mostkowska and G. Rousseau , “Repurposing of rituximab Biosimilars to Treat B Cell Mediated Autoimmune Diseases,” The FASEB Journal 38, no. 5 (2024): 23536, 10.1096/fj.202302259RR. 38470360

[33] E. Vecchio , R. Marino , S. Mimmi , et al., “Enhanced Pro‐apoptotic Activity of Rituximab through IBTK Silencing in Non‐Hodgkin Lymphoma B‐cells,” Frontiers in Oncology 14 (2024): 1339584, 10.3389/fonc.2024.1339584. 38371626 PMC10869532

[34] P. Guglielmi and F. Davi , “Expression of a Novel Type of Immunoglobulin C λ Transcripts in human Mature B Lymphocytes Producing χ Light Chains,” European Journal of Immunology 21, no. 2 (1991): 501–508, 10.1002/eji.1830210237. 1900243

[35] E. Verschueren , B. Husain , K. Yuen , et al., “The Immunoglobulin Superfamily Receptome Defines Cancer‐Relevant Networks Associated with Clinical Outcome,” Cell 182, no. 2 (2020): 329–344.e19, 10.1016/j.cell.2020.06.007. 32589946

[36] S. E. Degn and P. Tolar , “Towards a Unifying Model for B‐cell Receptor Triggering,” Nature Reviews Immunology 25, no. 2 (2025):77–91, 10.1038/s41577-024-01073-x. 39256626

[37] B. Kloo , D. Nagel , M. Pfeifer , et al., “Critical Role of PI3K Signaling for NF‐κB–dependent Survival in a Subset of Activated B‐cell–Like Diffuse Large B‐cell Lymphoma Cells,” Proceedings of the National Academy of Sciences U S A 108, no. 1 (2011): 272–277, 10.1073/pnas.1008969108. PMC3017191 21173233

[38] Y. Lee , M. Chittezhath , V. Andre , et al., “Protumoral Role of Monocytes in human B‐cell Precursor Acute Lymphoblastic Leukemia: Involvement of the Chemokine CXCL10,” Blood 119, no. 1 (2012): 227–237, 10.1182/blood-2011-06-357442. 22058116

[39] N. Karin , “CXCR3 Ligands in Cancer and Autoimmunity, Chemoattraction of Effector T Cells, and beyond,” Front Immunol 11 (2020): 976. 32547545 10.3389/fimmu.2020.00976 PMC7274023

[40] M. B. Watowich and M. R. Gilbert , “T Cell Exhaustion in Malignant Gliomas,” Trends in Cancer 9, no. 4 (2023): 270–292, 10.1016/j.trecan.2022.12.008. 36681605 PMC10038906

[41] L. Menzel , M. Zschummel , T. Crowley , et al., “Lymphocyte Access to Lymphoma Is Impaired by High Endothelial Venule Regression,” Cell Reports 37, no. 4 (2021): 109878, 10.1016/j.celrep.2021.109878. 34706240 PMC8567313

[42] L. Menzel , U. E. Höpken , and A. Rehm , “Angiogenesis in Lymph Nodes Is a Critical Regulator of Immune Response and Lymphoma Growth,” Frontiers in Immunology 11 (2020): 591741, 10.3389/fimmu.2020.591741. 33343570 PMC7744479

[43] L. Cascione , A. Rinaldi , A. Bruscaggin , et al., “Novel Insights into the Genetics and Epigenetics of MALT Lymphoma Unveiled by next Generation Sequencing Analyses,” Haematologica 104, no. 12 (2019): e558–e561, 10.3324/haematol.2018.214957. 31018978 PMC6959164

[44] A. Clipson , M. Wang , L. de Leval , et al., “KLF2 mutation Is the Most Frequent Somatic Change in Splenic Marginal Zone Lymphoma and Identifies a Subset with Distinct Genotype,” Leukemia 29, no. 5 (2015): 1177–1185, 10.1038/leu.2014.330. 25428260

