# Supplementary material for: Decoding IGLL5 Mutation‐Mediated BCR Signaling: A Novel Mechanism of CD8+ T Cell Exhaustion and Ocular MALT Lymphoma Progression
Source: Adv Sci (Weinh). 2026 May 12:e18780. Online ahead of print. doi: 10.1002/advs.202518780 (PMC13335846; doi:10.1002/advs.202518780)
Supplement: Supplementary file 1 — Supporting File 1: advs75628‐sup‐0001‐SuppMat.docx. [file ADVS-9999-e18780-s001.docx]

Supplemental Information

**Decoding IGLL5 mutation-mediated BCR signaling: a novel mechanism of CD8⁺ T cell exhaustion and ocular MALT lymphoma progression**

Index

| Supplementary Methods | page 2 |
| --- | --- |
| Supplementary Figure 1 | page 7 |
| Supplementary Figure 2 | page 9 |
| Supplementary Figure 3 | page 11 |
| Supplementary Figure 4 | page 13 |
| Supplementary Figure 5 | page 15 |
| Supplementary Figure 6 | page 17 |
| Supplementary Figure 7 | page 19 |
| Supplementary Figure 8 | page 21 |
| Supplementary Figure 9 | page 22 |
| Supplementary Table 1 | page 23 |
| Supplementary Table 2 | page 24 |
| Supplementary Table 3 | page 25 |
| Supplementary Table 4 | page 26 |
|  |  |

# Supplemental Methods

## Somatic variant validation by Sanger sequencing

Genomic DNA was isolated from tumor tissues and paired blood samples using the DNeasy Tissue and Blood kit (Qiagen, Valencia, CA), following previous observations[1]. DNA concentrations were quantified using a NanoDrop 2000 spectrophotometer (ThermoFisher Scientific, Japan), yielding concentrations between 50 and 2800 ng/μL with an A260/280 ratio between 1.8 and 2.0. For Sanger sequencing, 1 μg of DNA was used as a template in PCR amplifications using Green Taq Mix (Vazyme, China) under the recommended cycling conditions. Primers, designed with Primer3 v4.0.0 (<http://bioinfo.ut.ee/primer3/>), were synthesized by Tsingke Biotechnology Co. (Nanjing, China) to generate amplicons of 100–200 base pairs. Tsingke Biotechnology Co. then sequenced the PCR products, and sequence analysis was performed with Chromas software (Technelysium, Australia) to confirm the presence of somatic mutations.

## Co-immunoprecipitation (Co-IP) assay

Co-IP assays were conducted to confirm protein-protein interactions using the IP/Co-IP Kit (Thermo Fisher Scientific). Cells were first lysed in lysis buffer, and the resulting lysates were incubated overnight at 4°C with a specific antibody targeting the protein of interest. The immune complexes were then combined with A/G-agarose beads and incubated for 1 hour at room temperature. After incubation, the beads were washed twice with lysis buffer to eliminate any non-specifically bound proteins. The final complexes were dissolved in 1× SDS lysis buffer, denatured at 95°C for 10 minutes, and subjected to SDS-PAGE to separate the proteins by size. Detection of the immunoprecipitated protein was performed via Western blot analysis.

## Glutathione S-transferase (GST)-fusion pull-down assay

GST-fusion pull-down assay was analyzed according to the previous study[2]. FLAG-tagged IGLL5 expression plasmids were transfected into *E. coli* and purified using a Ni²⁺-NTA-chelating column (Thermo Fisher Scientific, MA, USA). For GST-fusion assays, 50 mg of GST-CD79A, GST-CD79B or GST protein was pre-equilibrated with glutathione Sepharose beads (Sigma, MO, USA) and subsequently incubated with 20 mg of purified FLAG-IGLL5. After overnight incubation, unbound proteins were washed away, and the remaining bound proteins were extracted from the beads using SDS sample buffer. These complexes were then separated by SDS-PAGE and detected by Western blot with anti-FLAG and GST antibodies to confirm interaction.

## Cell proliferation assay

Cell proliferation was evaluated using the Cell Counting Kit-8 (CCK-8, Dojindo, Japan) according to the manufacturer’s protocol. Briefly, transfected cells were plated in 96-well plates at a density of 1 × 10³ cells per well in 100 μl of medium and incubated at 37°C in a 5% CO₂ atmosphere. After the desired incubation period, 10 μl of CCK-8 solution was added to each well, followed by an additional 2-hour incubation at 37 °C. Absorbance was then measured at 450 nm to determine cell proliferation.

## Flow cytometry

Flow cytometry was employed to evaluate cell cycle distribution, apoptosis, and T-cell exhaustion, with data analyzed using FlowJo software.

For the cell cycle assay, treated cells (1 × 10⁶) were fixed in 75% ethanol and stored at –20 °C overnight. The fixed cells were then washed three times and stained with propidium iodide (PI) buffer according to the manufacturer’s protocol (Cell Cycle Analysis Kit, Beyotime, Shanghai, China).

To assess apoptosis, transfected cells were either left untreated or pre-incubated with rituximab (20 mg/mL; anti-CD20 chimeric monoclonal antibody, MCE, USA) for 1 h. Apoptotic activity was subsequently measured using the Annexin V-APC/7-AAD Apoptosis Detection Kit (KeyGEN, Jiangsu, China).

For analysis of tumor-infiltrating T cells, single-cell suspensions were prepared from subcutaneous tumors and subjected to flow cytometry. Live lymphocytes were gated based on forward and side scatter parameters, followed by the identification of CD45⁺ cells. Within this population, CD3⁺ T cells were selected, and CD8⁺ T cells were subsequently gated. T-cell exhaustion was evaluated by assessing co-expression of programmed cell death protein 1 (PD-1) and T-cell immunoglobulin and mucin domain-containing protein 3 (TIM-3), with exhausted CD8⁺ T cells defined as CD8⁺PD-1⁺TIM-3⁺. Gating strategies were applied consistently across all experimental groups, and representative plots were used to validate the approach.

## Histological analyses

Immunohistochemistry (IHC) and immunofluorescence (IF) analyses were performed on formalin-fixed, paraffin-embedded (FFPE) human tissue sections according to standard protocols. For IHC, 5-μm sections were deparaffinized and rehydrated before antigen retrieval. Sections were then blocked and incubated with primary antibodies overnight at 4 °C. Following incubation with HRP-conjugated secondary antibodies, staining was visualized. Staining intensity scores (0-3) and percentage of positive staining were quantified using ImageJ with the IHC Profiler plugin, and a staining index was calculated by multiplying the intensity score by the staining percentage. For IF, tissues were processed similarly, followed by permeabilization with 0.3% Triton X-100 and blocking with 1% BSA. Sections were then incubated with primary antibodies at 4 °C overnight, followed by fluorescent dye-conjugated secondary antibodies. Nuclei were counterstained with DAPI, and images were captured using a fluorescence microscope (Thunder Image, LEICA, Munich, Germany). Quantitative analysis of mean fluorescence intensity (MFI) and colocalization was performed using ImageJ.

## Elisa

To quantify protein levels, ELISA assays were conducted following the manufacturer’s protocols. Cell culture supernatants were collected from 96-well plates, and IGLL5, along with cytokines and chemokines such as CXCL10 and CXCL11, were measured using human-specific ELISA kits (Proteintech, China) on a Biotech microplate reader (ThermoFisher Scientific, USA). The concentrations of these proteins were calculated from Optical Density (OD) values obtained at 450 nm, based on a standard curve generated from known concentrations.

## CD8^+^ T Cell Migration Assay

Human CD8+ T cells were first isolated from peripheral blood mononuclear cells using a Human CD8+ T Cell Isolation Kit (Biolegend, USA) with magnetic bead separation. The cells were then activated with ImmunoCult Human CD3/CD28/CD2 T cell activator and recombinant human IL-2. For the migration assay, a 24-well transwell system with a 3 µm pore size polycarbonate membrane (Corning, USA) was used, as mentioned previously[3]. In each assay, 600 µL of supernatant from various transfected Raji and OCI-LY19 cell lines was added to the lower chamber, while 1 × 10^5^ isolated CD8+ T cells were placed in the upper chamber. Following a 6-hour incubation at 37 °C, migrated T cells in the lower chamber were collected and counted by flow cytometry.

## Animal experiments

Five-week-old female BALB/c-nude and BALB/c mice were purchased from GemPharmatech Co., Ltd. (China) and maintained in a specific pathogen-free facility. All animal procedures were performed in accordance with protocols approved by the Institutional Animal Care and Use Committee of the Chinese Academy of Medical Sciences (NO. 2502025).

To evaluate the effect of BTK inhibition *in vivo*, OCI-LY19 or RAJI cells (1 × 10⁷) transfected with either WT or mutant IGLL5 plasmids were suspended in diluted Matrigel (354248, Corning, USA) and injected subcutaneously into BALB/c-nude mice (n = 20 per xenograft model). One week after inoculation, mice were randomly assigned to receive either PBS or ibrutinib (10 mg/kg, intraperitoneally, once weekly). Each group included five mice. Following three weeks of treatment, mice were euthanized, and xenograft tumors were harvested for IHC staining of Ki67 and Cleaved Caspase-3.

To establish the syngeneic A20 tumor model, A20 cells were injected subcutaneously into BALB/c mice (n = 30). One week later, mice were randomized into three treatment groups: PBS, rituximab (RTX, 10 mg/kg, intraperitoneally, once weekly), or RTX (10 mg/kg, intraperitoneally, once weekly) combined with ibrutinib (10 mg/kg, intraperitoneally, once weekly). Each group included five mice. Tumor burden was monitored by in vivo imaging at day 21, and mice were euthanized at day 28. Tumor tissues were collected for IHC staining of Ki67 and Cleaved Caspase-3 (n = 5 per group). Tumor volume and body weight were recorded every three days, with tumor volume calculated as: Tumor volume = length×width^2^/2

# Supplemental Figures


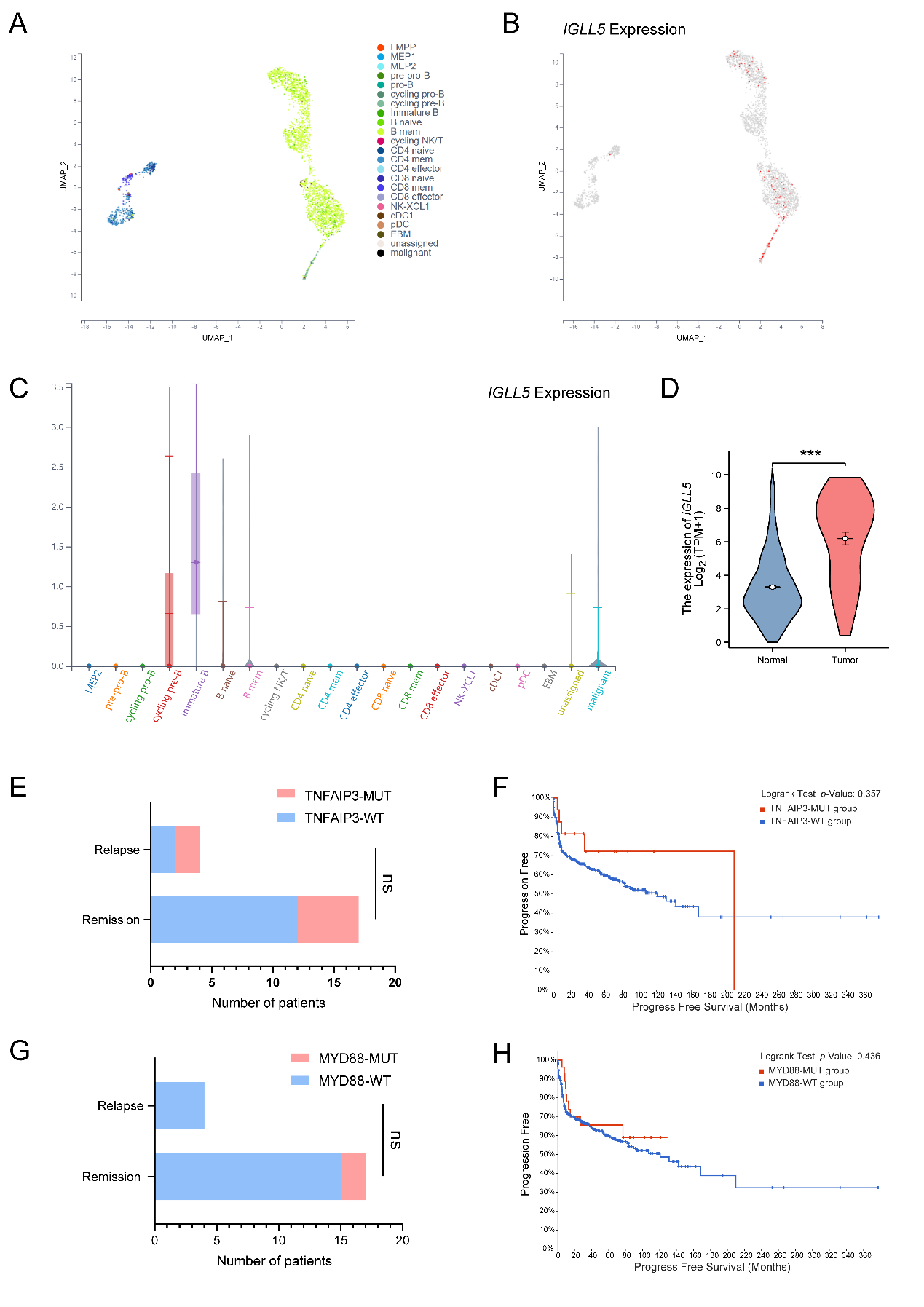


## Figure S1. IGLL5 is a potential biomarker in B-cell malignancies.

(**A**) Uniform Manifold Approximation and Projection (UMAP) plot displaying clustering results colored by the major cellular compartments in the GSE115795 dataset. (**B**) Feature plots showing the distribution of IGLL5 expression across different cellular compartments in the dataset. (**C**) Histogram illustrating IGLL5 expression levels within specific cellular compartments. (**D**) TCGA diffused large B-cell lymphoma (DLBCL) data indicate the concordance of mRNA pairs is significantly higher in DLBCL than in the normal controls. Statistical significance was determined using two-tailed unpaired Student’s *t*-test. (**E, G**) Association between somatic *TNFAIP3*(**E**) and *MYD88*(**G**) mutations and response to a rituximab-containing postoperative treatment in the OAML cohort. Statistical differences were measured using Fisher's exact test. (**F, H**) Kaplan-Meier plot showing the progression free survival (PFS) differences between *TNFAIP3*-mutant(**F**) or *MYD88*-mutant(**H**) and wild-type patients based on the Cancer Genomics database. Survival differences were assessed using the log-rank test. Statistical significance is denoted as ^ns^*p*<0.001, ^***^*p*<0.001. WT: wild-type. MUT: mutant.


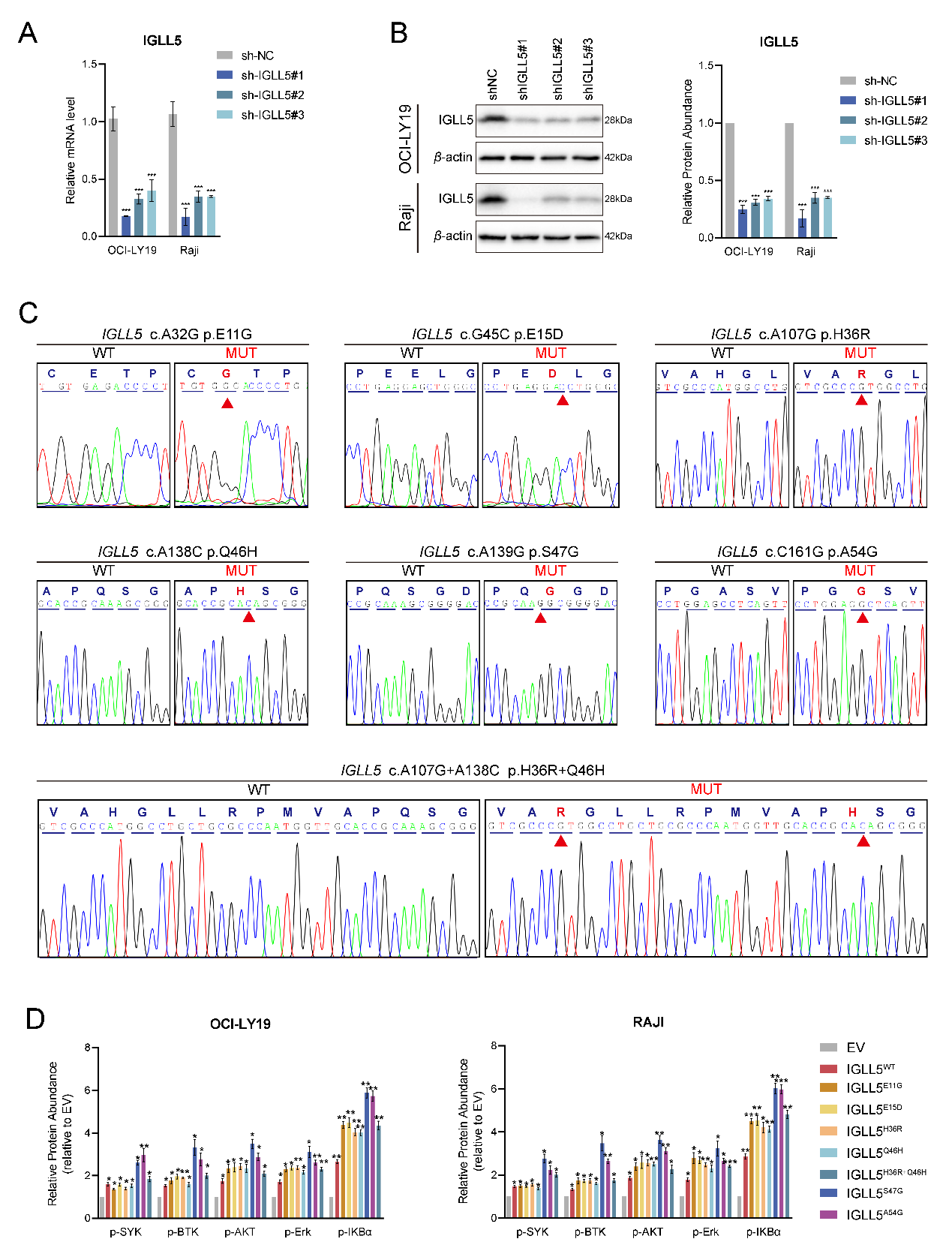


## Figure S2. Construction of IGLL5 mutant cell lines.

(**A, B**) RT-qPCR (**A**) and western blot (**B**) were used to measure the knockdown efficiency of endogenous IGLL5 in OCI-LY19 and Raji cell lines. Statistical significance was determined using two-tailed unpaired Student’s t-test. (**C**) Sanger sequencing was used to confirm transduction with lentiviruses containing the empty vector (EV), wild-type (WT), and respective mutant (p.E11G, p.E15D, p.H36R, p.Q46H, p.S47G, p.A54G and p.H36R+Q46H) IGLL5 in OCI-LY19 and Raji cell lines. (**D**) Quantification of phosphorylated levels in multiple signaling pathways in OCI-LY19 and Raji cells transduced with different IGLL5 mutants. Total protein levels were used as a control for phosphorylated proteins, and the ratios of phosphorylated expression were normalized to the EV control. Statistical significance was determined by one-way ANOVA followed by Tukey’s multiple-comparisons test. All data are presented as the means ± SD of three biologically independent experiments. Statistical significance is denoted as ^**^*p* <0.01, ^***^*p* <0.001. EV: empty vector. WT: wild-type.


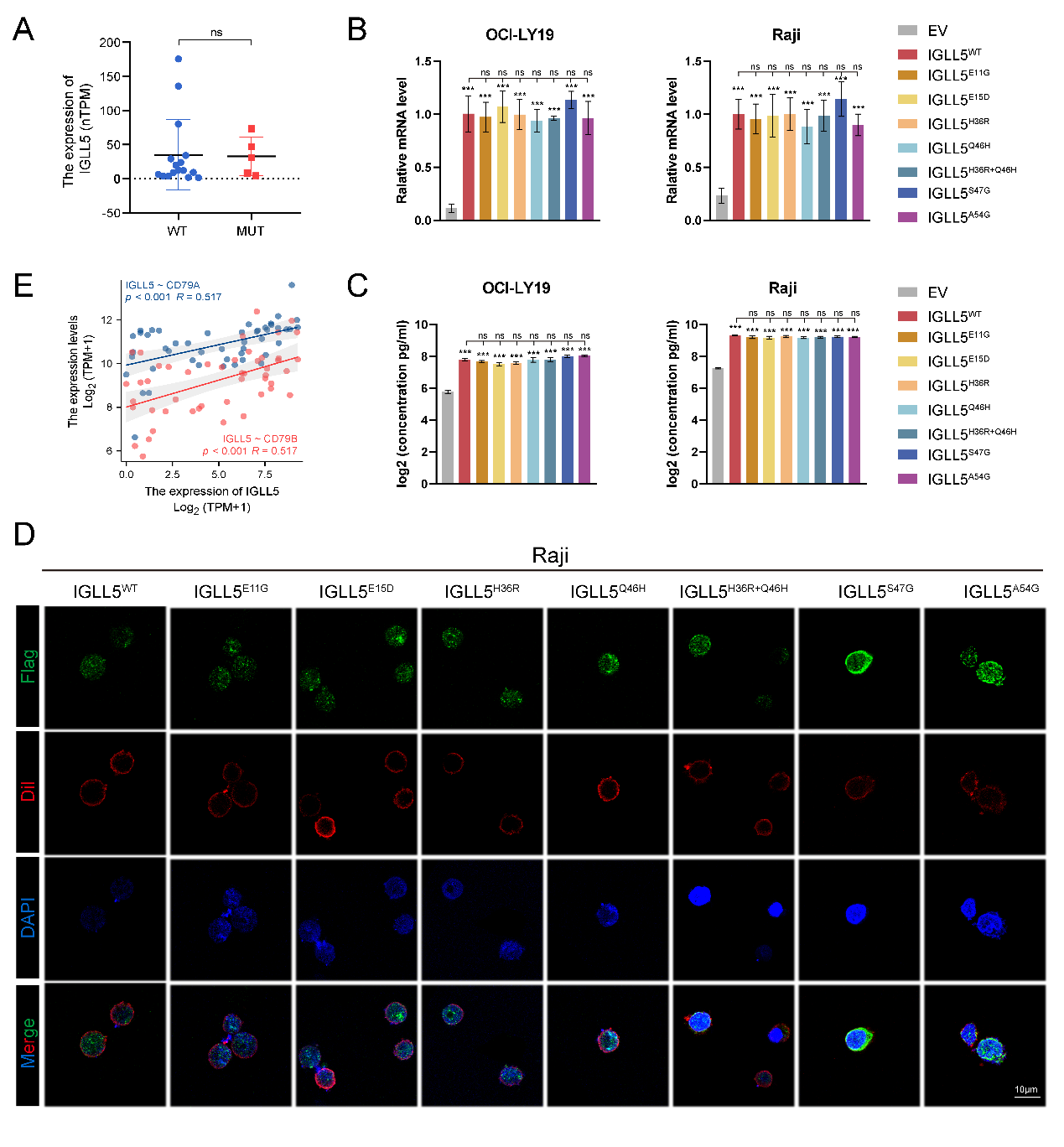


## Figure S3. IGLL5 mutations affect protein localization rather than expression levels.

(**A**) Comparison of *IGLL5* mRNA levels between IGLL5-mutant and wild-type patients in the OAML cohort. Statistical significance was determined using two-tailed unpaired Student’s *t*-test. (**B, C**) RT-qPCR analysis(**B**) and ELISA detection(**C**) of IGLL5 expression were conducted in OCI-LY19 and Raji cell lines transduced with different *IGLL5* mutants, using *ACTB* as an input control. Statistical significance was determined by one-way ANOVA followed by Tukey’s multiple-comparisons test, with significance indicated below the horizontal line normalized to the EV control and above the line normalized to the WT control. (**D**) Immunofluorescence analysis of the localization of WT IGLL5 and various mutants in Raji cells transfected with FLAG-tagged plasmids. Cells were fixed and stained with an anti-FLAG antibody, followed by a goat-anti-rabbit IgG-AF488 antibody (green). Nuclei were counterstained with DAPI (blue), and cell membranes were stained with Dil perchlorate (red). (**E**) Correlation analysis of *IGLL5* expression with *CD79A* and *CD79B* in the TCGA-DLBCL cohort. Spearman’s rank correlation test was used to assess the relationship. All data are presented as the means ± SD of three independent experiments. Statistical significance is denoted as ^ns^*p* >0.05, ^***^*p* <0.001. EV: empty vector. MUT: mutant. WT: wild-type.


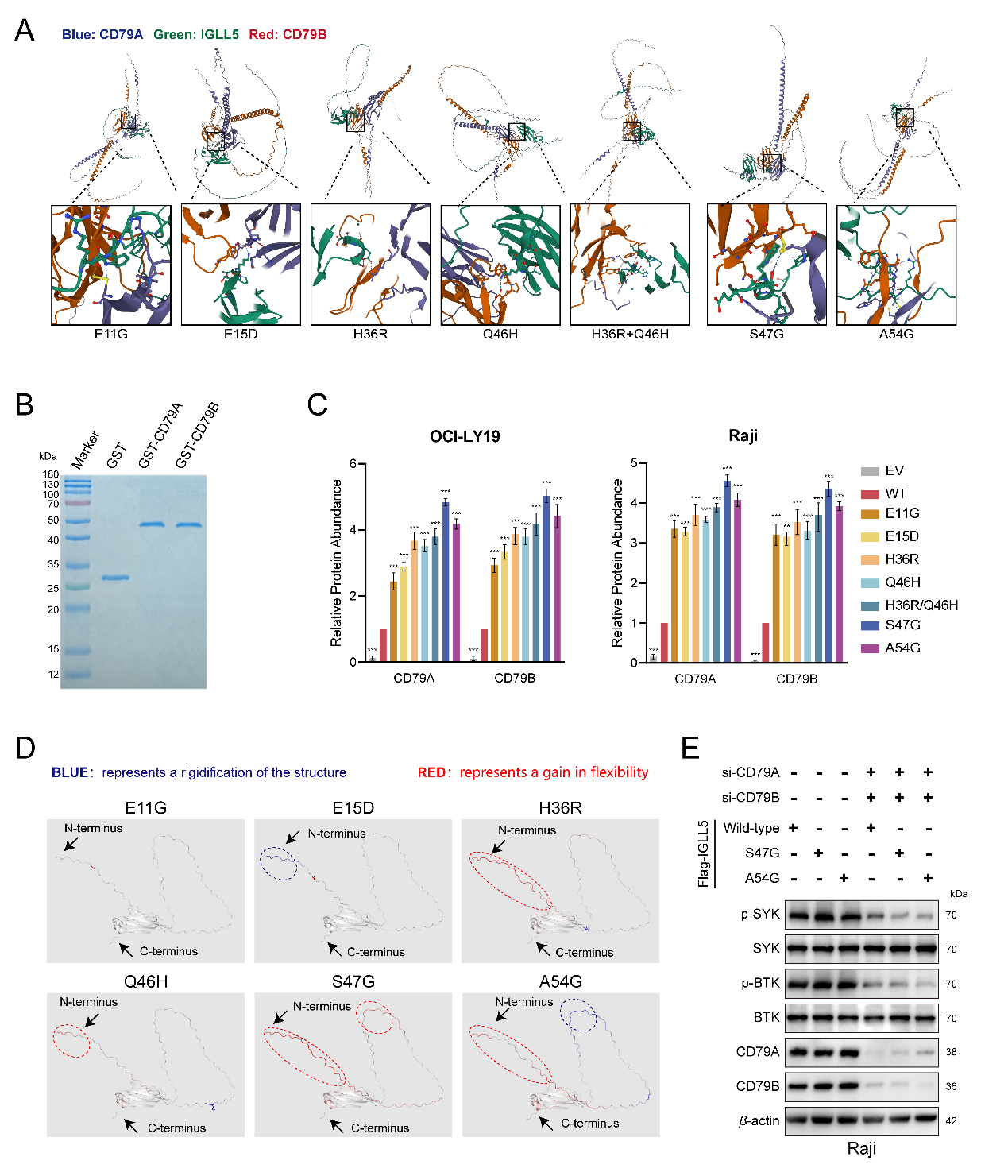


## Figure S4. Mutations in IGLL5 increase its binding affinity to the CD79A/CD79B complex.

(**A**) The 3D structures of CD79A, CD79B, and IGLL5 carrying various mutations were predicted using AlphaFold3. The results showed that all IGLL5 mutants could interact with CD79A and CD79B. (**B**) Glutathione S-transferase (GST) fusions of CD79A and CD79B were expressed in E. coli and assayed. (**C**) Quantitative analysis of Co-IP and Western blot assays in OCI-LY19 and Raji cells testing the endogenous interactions between WT or mutant IGLL5 and CD79A, as well as CD79B. The protein levels of mutant IGLL5 in the Co-IP assays were normalized to the WT control. Statistical significance was determined by one-way ANOVA followed by Tukey’s multiple-comparisons test. (**D**) Visual representation of the change in vibrational entropy energy between WT and missense mutations generated using DynaMut (<http://biosig.unimelb.edu.au/dynamut/>). (**E**) Western blot analysis showing expression of CD79A, CD79B, SYK, phospho-SYK, BTK, and phospho-BTK in Raji cell expressing wild-type (WT), S47G-, or A54G-mutant IGLL5 following knockdown of CD79A and/or CD79B. Representative immunoblots from three biologically independent experiments are shown. All data are presented as the means ± SD of three independent experiments. Statistical significance is denoted as ^**^*p* <0.01, ^***^*p* <0.001. EV: empty vector. WT: wild-type.

**
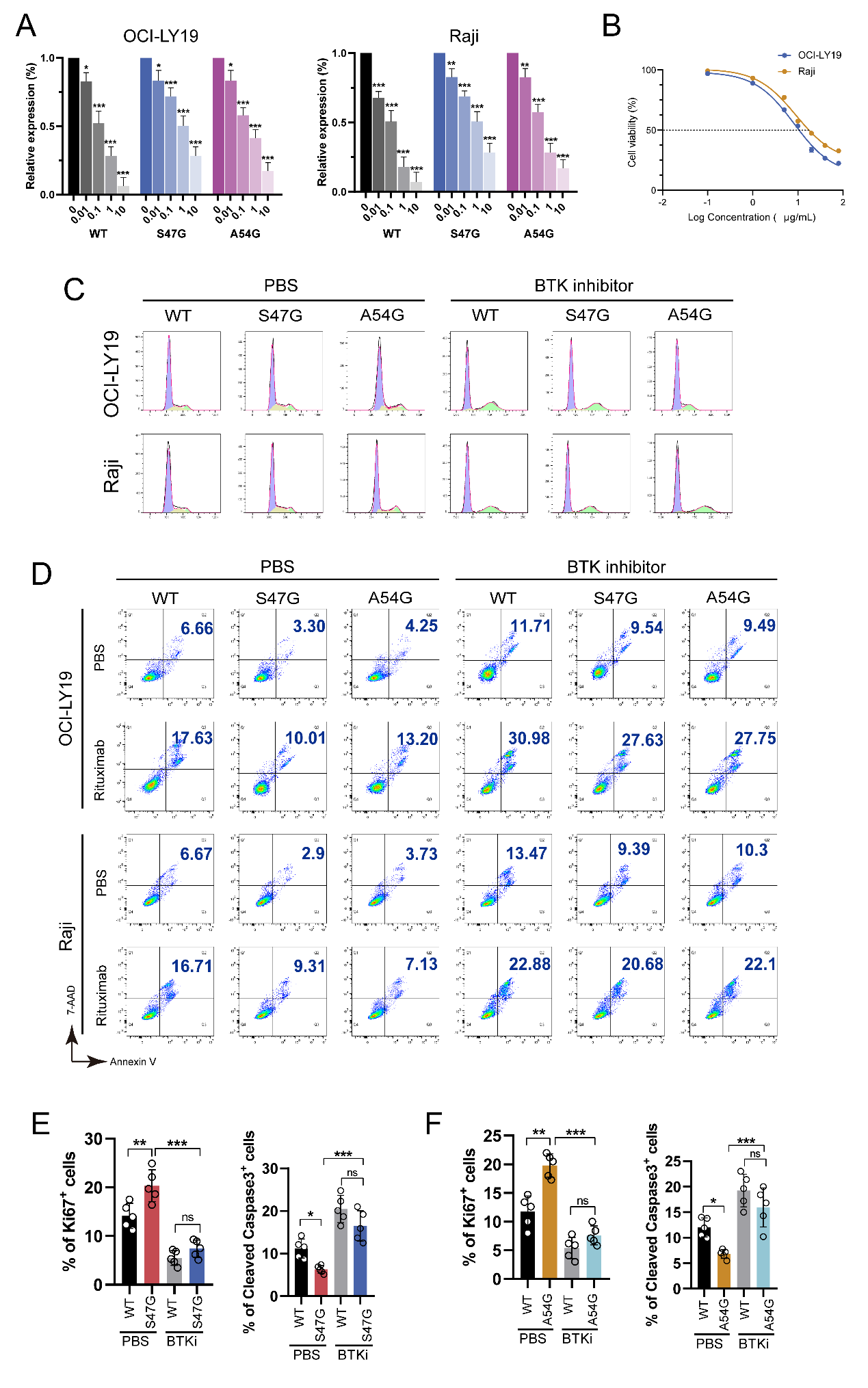
**Figure S5. **BTKi inhibits BCR-related signaling and attenuates IGLL5 mutant-associated phenotypes in lymphoma cells.**

(**A**) Quantification of phospho-BTK and total BTK protein levels by Western blot in wild-type, S47G-, and A54G-mutant OCI-LY19 and Raji cells treated with increasing concentrations of ibrutinib for 2 hours. Data are presented as mean ± SD from three biologically independent experiments. Statistical significance was determined by one-way ANOVA followed by Tukey’s multiple-comparisons test. (**B**) Cell cycle distribution in wild-type and mutant (S47G, A54G) OCI-LY19 and Raji cells treated with or without BTKi. Data are presented as mean ± SD from three biologically independent experiments. Statistical significance was determined by one-way ANOVA followed by Tukey’s multiple-comparisons test. (**C**) Apoptosis analysis of OCI-LY19 and Raji cells expressing wild-type or mutant (S47G, A54G) IGLL5, treated with or without BTKi in the presence or absence of RTX. Data are presented as mean ± SD from three biologically independent experiments. Statistical significance was determined by one-way ANOVA followed by Tukey’s multiple-comparisons test. (**D**, **E**) Quantitative analysis of Ki67 and cleaved Caspase3 expression by immunohistochemistry in xenograft tumor tissues. Quantification is presented as mean ± SD (n = 5 mice per group). Statistical significance was determined by one-way ANOVA followed by Tukey’s multiple-comparisons test. Statistical significance is denoted as ^ns^*p* >0.05, ^*^*p* <0.05, ^**^*p* <0.01, ^***^*p* <0.001.


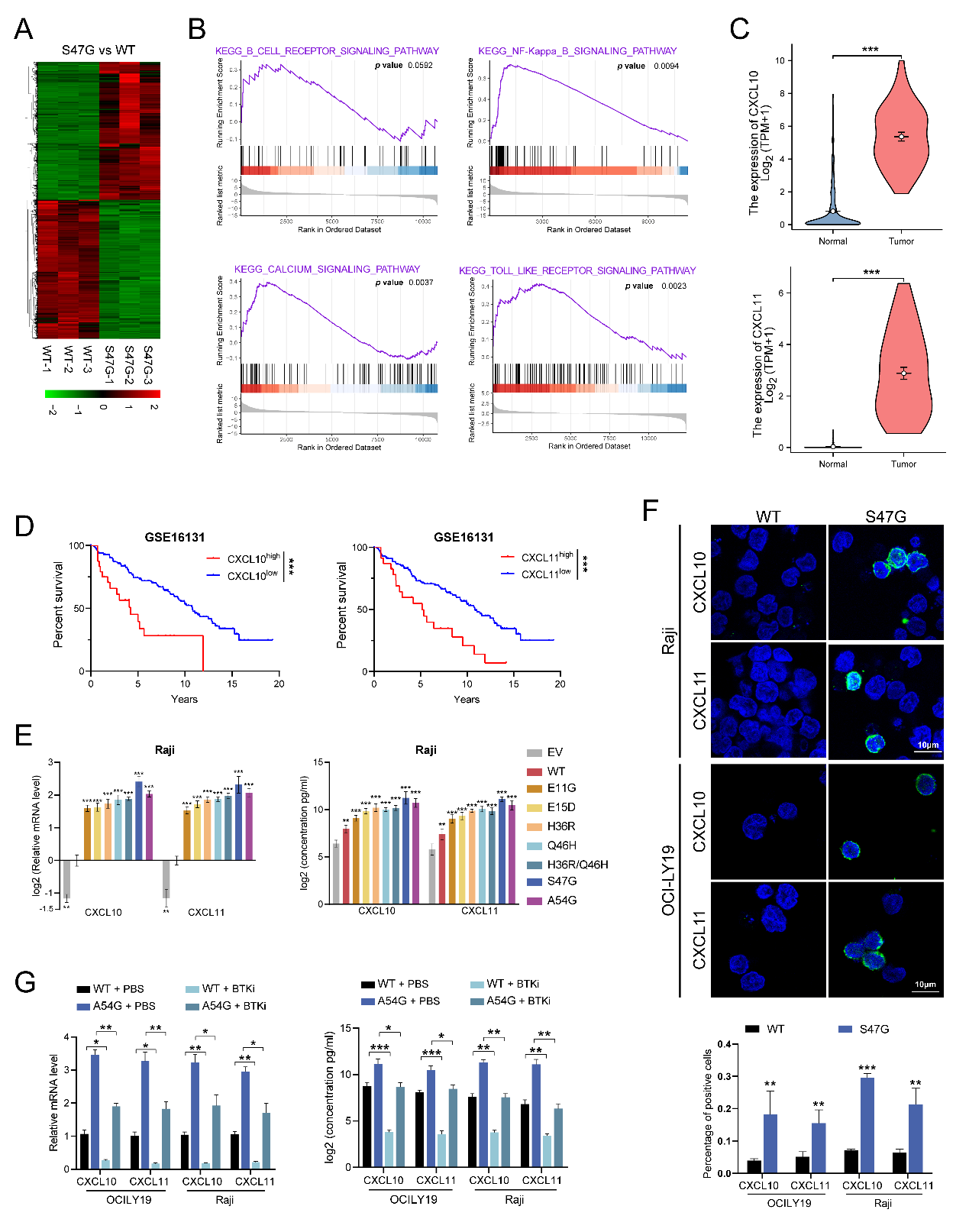


## Figure S6. IGLL5 mutations increase CXCL10 and CXCL11 levels by activating the B-cell receptor (BCR) pathway.

(**A**) Heatmap of transcriptome data from OCI-LY19 cells transfected with IGLL5 S47G versus IGLL5 WT plasmid. (**B**) GSEA gene set enrichment analysis indicated that significantly enriched gene sets were associated with downstream of the BCR pathway in OCI-LY19 cells transfected with IGLL5 S47G versus IGLL5 WT plasmid. (**C**) TCGA-DLBCL data showing significantly higher mRNA expression of CXCL10 and CXCL11 in DLBCL compared with normal controls. Statistical significance was determined using two-tailed unpaired Student’s *t*-test. (**D**) Kaplan–Meier plots showing overall survival stratified by CXCL10 (left) or CXCL11 (right) expression in the GSE16131 dataset. Survival differences were assessed using the log-rank test. (**E**) RT-qPCR and ELISA analyses of CXCL10 and CXCL11 expression in Raji cells transduced with the indicated IGLL5 mutants. ACTB (β-actin) was used as the internal control for RT-qPCR. Statistical significance was determined by one-way ANOVA followed by Tukey’s multiple-comparisons test, with significance normalized to the WT control. (**F**) Immunofluorescence analysis of CXCL10 and CXCL11 expression in Raji and OCI-LY19 cells transfected with IGLL5 S47G or WT IGLL5 (upper). Quantification of CXCL10⁺ and CXCL11⁺ cells is shown below. Statistical significance was determined using two-tailed unpaired Student’s t-test. (**G**) RT-qPCR and ELISA analyses of CXCL10 and CXCL11 expression in Raji and OCI-LY19 cells transduced with IGLL5 A54G or WT IGLL5, with or without ibrutinib treatment. Statistical significance was determined by one-way ANOVA followed by Tukey’s multiple-comparisons test. All data are presented as mean ± SD from three biologically independent experiments. ^*^*p* <0.05, ^**^*p* <0.01, ^***^*p* <0.001. EV: empty vector. WT: wild-type.


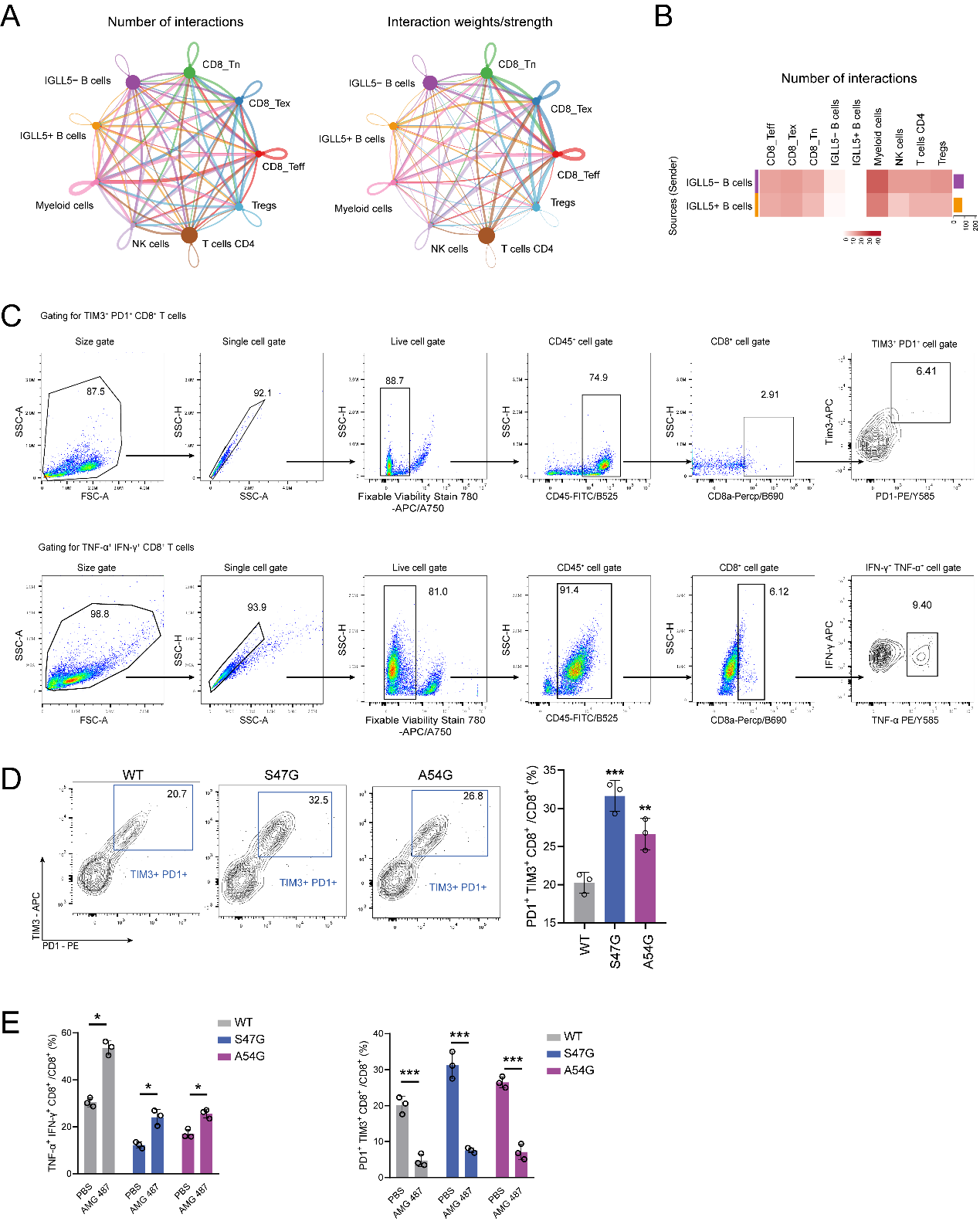


## Figure S7. Differential cell chat between IGLL5^+^ B cell and IGLL5- B cell.

(**A**) Cell-cell interaction map (left) and interaction strength network (right) from the GSE182434 dataset, showing the number of outgoing interactions at each node. (**B**) Differential interaction strengths of a cellular interactome between IGLL5^+^ B cell and IGLL5^-^ B cell types in the GSE182434 dataset. (**C**) Gating strategy and representative flow cytometry plots showing the identification of PD-1^+^TIM-3^+^CD8⁺ T-cell populations (upper) and INFγ^+^TNFα^+^CD8⁺ T-cell populations (lower). **(D)** Representative flow cytometry plots (left) showing TIM3^+^PD1^+^CD8⁺ T cells in the subcutaneous tumor model. Quantification (right) shows the proportion of TIM3^+^PD1^+^CD8⁺ T cells among total CD8⁺ T cells across experimental groups. Data are presented as mean ± SD (n = 3 mice per group). Statistical significance was determined by one-way ANOVA followed by Tukey’s multiple-comparisons test. All data are presented as the means ± SD of three independent experiments. **(E)** Quantification of PD-1^+^TIM-3^+^CD8⁺ T cells and INFγ^+^TNFα^+^CD8⁺ T cells among total CD8⁺ T cells across the indicated experimental groups. Data are presented as mean ± SD (n = 3 mice per group). Statistical significance was determined by one-way ANOVA followed by Tukey’s multiple-comparisons test. Statistical significance is denoted as ^ns^*p* >0.05, ^**^*p* <0.01, ^***^*p* <0.001.


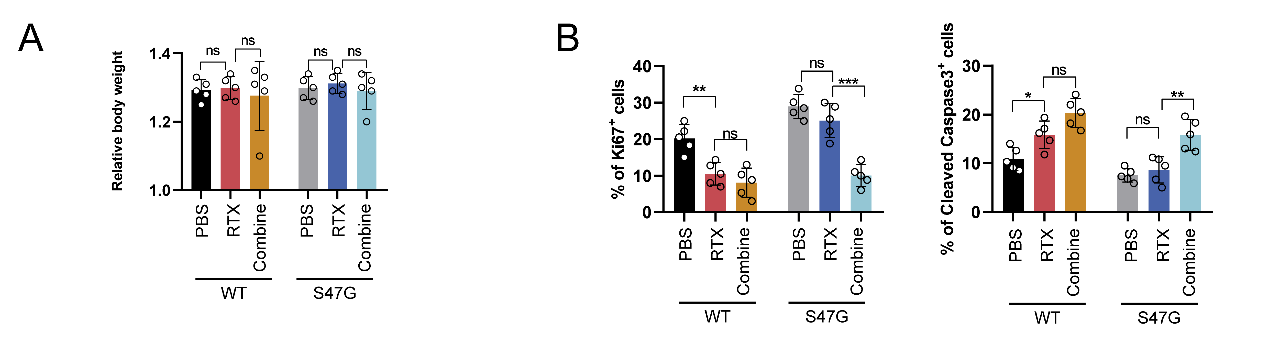


## Figure S8. Combined BTKi and RTX treatment shows enhanced antitumor efficacy in an IGLL5-mutant B-cell lymphoma model.

(**A**) Body weight dynamics of mice across treatment groups during the 28-day study period. Data are presented as mean ± SD (n = XX mice per group). Statistical significance was determined by two-way ANOVA followed by Tukey’s multiple-comparisons test. (**B**) Quantification of Ki67 and cleaved caspase-3 staining shown in Figure 8C. Data are presented as mean ± SD (n = 5 mice per group). Statistical significance was determined by one-way ANOVA followed by Tukey’s multiple-comparisons test. Statistical significance is denoted as ^ns^*p* >0.05, ^*^*p* <0.05, ^**^*p* <0.01, ^***^*p* <0.001.


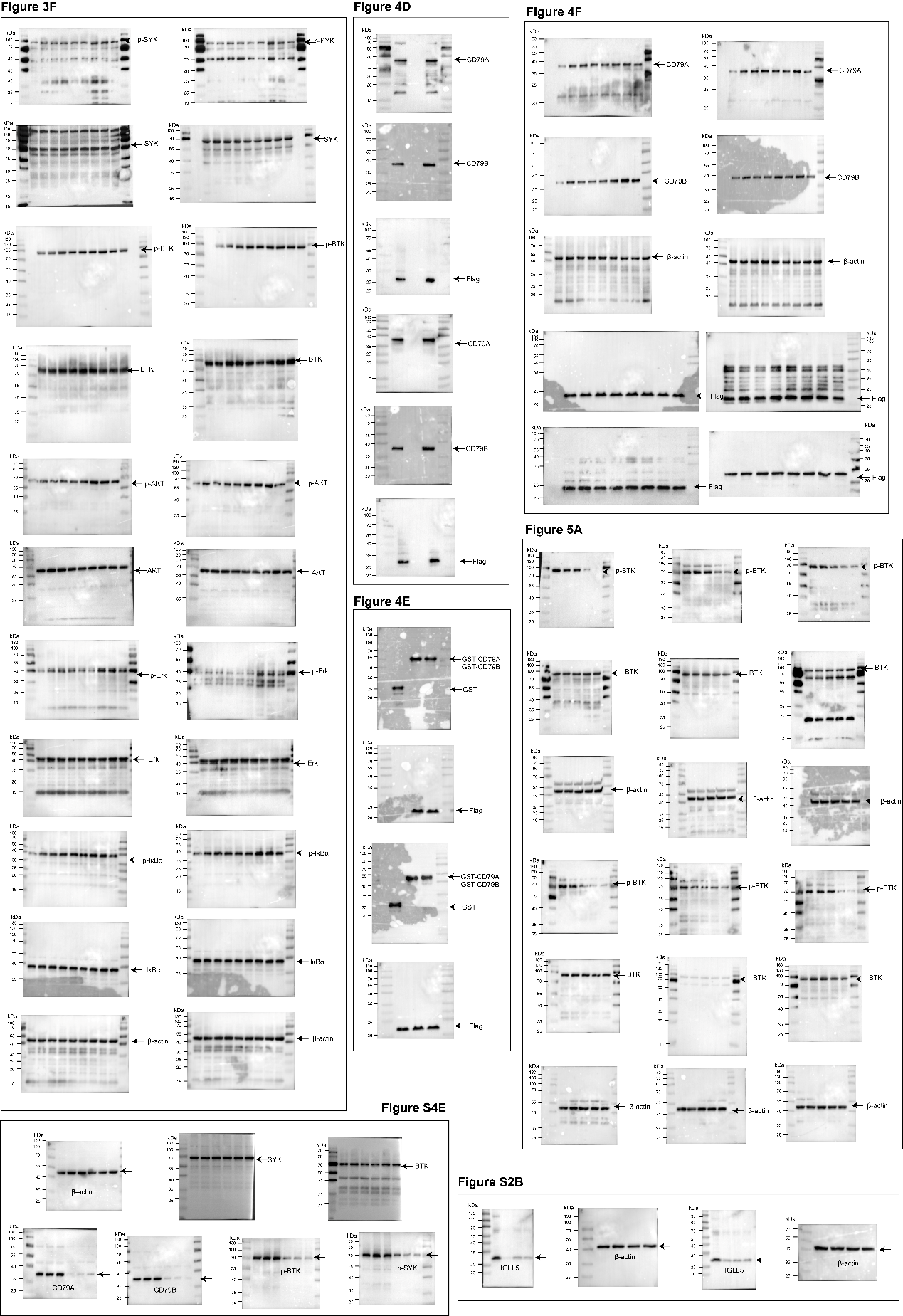


**Figure S9. Uncropped western blot images.**

# Supplemental Tables

## Table S1. Baseline Data Table Based on CXCL10 Expression Analysis

| Characteristics | Low expression of CXCL10 | High expression of CXCL10 | *p* value |
| --- | --- | --- | --- |
| n | 24 | 24 |  |
| Clinical stage, n (%) |  |  | 0.016 |
| Stage I | 7 (16.7%) | 1 (2.4%) |  |
| Stage II | 10 (23.8%) | 7 (16.7%) |  |
| Stage III | 0 (0%) | 5 (11.9%) |  |
| Stage IV | 5 (11.9%) | 7 (16.7%) |  |
| Primary therapy outcome, n (%) |  |  | 0.010 |
| CR | 22 (47.8%) | 13 (28.3%) |  |
| PD&PR&SD | 2 (4.3%) | 9 (19.6%) |  |

CR: Complete Response. PR: Partial Response. SD: Stable Disease. PD: Progressive Disease

## Table S2. Baseline Data Table Based on CXCL11 Expression Analysis

| Characteristics | Low expression of CXCL11 | High expression of CXCL11 | *p* value |
| --- | --- | --- | --- |
| n | 24 | 24 |  |
| Clinical stage, n (%) |  |  | 0.023 |
| Stage I | 7 (16.7%) | 1 (2.4%) |  |
| Stage II | 9 (21.4%) | 8 (19%) |  |
| Stage III | 0 (0%) | 5 (11.9%) |  |
| Stage IV | 6 (14.3%) | 6 (14.3%) |  |
| Primary therapy outcome, n (%) |  |  | 0.010 |
| CR | 22 (47.8%) | 13 (28.3%) |  |
| PD&PR&SD | 2 (4.3%) | 9 (19.6%) |  |

CR: Complete Response. PR: Partial Response. SD: Stable Disease. PD: Progressive Disease

## Table S3. Primer sequences used in the study

| **Primer name** | | **Primer sequences** |
| --- | --- | --- |
| *IGLL5* | Forward Primer | AGTCACTGTGTTATGTCTTCGGA |
|  | Reverse Primer | TCTGTGGGACTTCCACTGCT |
| *CXCL10* | Forward Primer | GTGGCATTCAAGGAGTACCTC |
|  | Reverse Primer | TGATGGCCTTCGATTCTGGATT |
| *CXCL11* | Forward Primer | GACGCTGTCTTTGCATAGGC |
|  | Reverse Primer | GGATTTAGGCATCGTTGTCCTTT |
| *ACTB* | Forward Primer | CATGTACGTTGCTATCCAGGC |
|  | Reverse Primer | CTCCTTAATGTCACGCACGAT |
| *IGLL5-plasmids* for PCR | Forward Primer | AGGTCGACTCTAGAGGATCCCGCCACCATGAGACCCAAGACAGGCCAAG |
|  | Reverse Primer | TCCTTGTAGTCCATGGATCCTGAACATTCTGTAGGGGCCACTGTCTTCTC |

## Table S4. List of primary antibodies

| **Name** | **Manufacturer** | **Catalog** | **Application** | **Dilution** |
| --- | --- | --- | --- | --- |
| Anti-IGLL5 antibody | Invitrogen | #PA5-49022 | Western blot | 1:1000 |
|  |  |  | Immunohistochemistry | 1:200 |
| Anti-Ki67 antibody | Proteintech | 27309-1-AP | Immunohistochemistry | 1:2000 |
| Anti-*β*-actin antibody | Proteintech | 81115-1-RR | Western blot | 1:5000 |
| Anti-Cleaved Caspase3 antibody | CST | #9661 | Immunohistochemistry | 1:400 |
| Anti-Bax antibody | Proteintech | 50599-2-Ig | Immunohistochemistry | 1:1000 |
| Anti-FLAG-tag antibody | Proteintech | 20543-1-AP | Western blot | 1:20000 |
|  |  |  | Immunoprecipitation | 2 µg/test |
|  |  |  | Immunofluorescence | 1:2000 |
| Anti-GST-tag antibody | CST | #2622 | Western blot | 1:1000 |
|  |  |  | Immunoprecipitation | 2 µg/test |
| Anti-IκBα antibody | CST | #4814 | Western blot | 1:1000 |
| Anti-p- IκBα antibody | CST | #2859 | Western blot | 1:1000 |
| Anti-Erk antibody | Abcam | ab50011 | Western blot | 1:10000 |
| Anti-p-Erk antibody | Abcam | ab184699 | Western blot | 1:10000 |
| Anti-AKT antibody | Proteintech | 60203-2-Ig | Western blot | 1:5000 |
| Anti-p-AKT antibody | Proteintech | 66444-1-Ig | Western blot | 1:2000 |
| Anti-CD79A antibody | Proteintech | 22349-1-AP | Western blot | 1:2000 |
| Anti-CD79B antibody | Proteintech | 21063-1-AP | Western blot | 1:1000 |
| Anti-CXCL10 antibody | Proteintech | 10937-1-AP | Immunofluorescence | 1:400 |
| Anti-CXCL11 antibody | Proteintech | 10707-1-AP | Immunofluorescence | 1:400 |
| Anti-CD8 antibody | Proteintech | 66868-1-Ig | Immunofluorescence | 1:400 |
| Anti-PD1 antibody | Abcam | ab52587 | Immunofluorescence | 1:100 |
| Anti-LAG3 antibody | Abcam | EPR20261 | Immunofluorescence | 1:100 |
| Fixable Viability Stain | BD Biosciences | 565388 | Flow cytometry | 0.5μl/test |
| Anti-CD45 antibody | BD Biosciences | 553079 | Flow cytometry | 1μl/test |
| Anti-CD3 antibody | BD Biosciences | 563024 | Flow cytometry | 1μl/test |
| Anti-CD8a antibody | BD Biosciences | 551162 | Flow cytometry | 1μl/test |
| Anti-TIM3 antibody | BD Biosciences | 119706 | Flow cytometry | 1μl/test |
| Anti-PD-1 antibody | BD Biosciences | 561788 | Flow cytometry | 1μl/test |
| Anti-Rabbit Secondary antibody | Proteintech | RGAR001 | Western blot | 1:10000 |
| Anti-Mouse Secondary antibody | Proteintech | SA00001-1 | Western blot | 1:10000 |

CST: Cell Signaling Technology

# Reference

1. Zhao A, Wu F, Wang Y, Li J, Xu W, Liu H: **Analysis of Genetic Alterations in Ocular Adnexal Mucosa-Associated Lymphoid Tissue Lymphoma With Whole-Exome Sequencing**. *Front Oncol* 2022, **12**:817635.

2. Zhao A, Zhou C, Li J, Wang Z, Zhu H, Shen S, Shao Q, Gong Q, Liu H, Chen X: **UBE2G2 inhibits vasculogenic mimicry and metastasis of uveal melanoma by promoting ubiquitination of LGALS3BP**. *Acta Pharmaceutica Sinica B* 2024.

3. Nagarsheth N, Peng D, Kryczek I, Wu K, Li W, Zhao E, Zhao L, Wei S, Frankel T, Vatan L *et al*: **PRC2 Epigenetically Silences Th1-Type Chemokines to Suppress Effector T-Cell Trafficking in Colon Cancer**. *Cancer Res* 2016, **76**(2):275-282.
